# The language of geometry: Fast comprehension of geometrical primitives and rules in human adults and preschoolers

**DOI:** 10.1371/journal.pcbi.1005273

**Published:** 2017-01-26

**Authors:** Marie Amalric, Liping Wang, Pierre Pica, Santiago Figueira, Mariano Sigman, Stanislas Dehaene

**Affiliations:** 1 Cognitive Neuroimaging Unit, CEA DSV/I2BM, INSERM, Université Paris-Sud, Université Paris-Saclay, NeuroSpin center, Gif/Yvette, France; 2 Sorbonne Universités, UPMC Univ Paris 06, IFD, Paris, France; 3 Collège de France, Paris, France; 4 Institute of Neuroscience, Key Laboratory of Primate Neurobiology, CAS Center for Excellence in Brain Science and Intelligence Technology, Chinese Academy of Sciences, Shanghai, China; 5 Instituto do Cérebro, Universidade Federal do Rio Grande do Norte, Natal, Brasil; 6 UMR 7023 Structures Formelles du Langage CNRS, Université Paris 8, Saint-Denis, France; 7 Department of Computer Science, FCEN, University of Buenos Aires and ICC-CONICET, Buenos Aires, Argentina; 8 Neuroscience Laboratory, Universidad Torcuato Di Tella, Buenos Aires, Argentina; Rutgers University, UNITED STATES

## Abstract

During language processing, humans form complex embedded representations from sequential inputs. Here, we ask whether a “geometrical language” with recursive embedding also underlies the human ability to encode sequences of spatial locations. We introduce a novel paradigm in which subjects are exposed to a sequence of spatial locations on an octagon, and are asked to predict future locations. The sequences vary in complexity according to a well-defined language comprising elementary primitives and recursive rules. A detailed analysis of error patterns indicates that primitives of symmetry and rotation are spontaneously detected and used by adults, preschoolers, and adult members of an indigene group in the Amazon, the Munduruku, who have a restricted numerical and geometrical lexicon and limited access to schooling. Furthermore, subjects readily combine these geometrical primitives into hierarchically organized expressions. By evaluating a large set of such combinations, we obtained a first view of the language needed to account for the representation of visuospatial sequences in humans, and conclude that they encode visuospatial sequences by minimizing the complexity of the structured expressions that capture them.

## Author Summary

The child’s acquisition of language has been suggested to rely on the ability to build hierarchically structured representations from sequential inputs. Does a similar mechanism also underlie the acquisition of geometrical rules? Here, we introduce a learning situation in which human participants had to grasp simple spatial sequences and try to predict the next location. Sequences were generated according to a “geometrical language” endowed with simple primitives of symmetries and rotations, and combinatorial rules. Analyses of error rates of various populations—a group of French educated adults, two groups of 5 years-old French children, and a rare group of teenagers and adults from an Amazonian population, the Mundurukus, who have limited access to formal schooling and a reduced geometrical lexicon—revealed that subjects’ learning indeed rests on internal language-like representations. A theoretical model, based on minimum description length, proved to fit well participants’ behavior, suggesting that human subjects “compress” spatial sequences into a minimal internal rule or program.

## Introduction

In the past decades, studies of sequence learning have outlined one possible mechanism by which complex mental representations are constructed out of simpler primitives: the human ability to extract complex nested structures from sequential inputs [1]. While non-human primates fail to show any systematicity in language learning [2], humans seem to be innately endowed with a quick grasp of complex embedded rules. At 8 months of age already, infants presented with a brief sequence of syllables readily extract recurrent 3-syllabic words [3,4], and by 12 months they understand how these words combine to form larger structures [5]. A similar ability to group consecutive items according to abstract regularities has also been demonstrated during the learning of visuomotor sequences by adults [6,7]. Children and adults are also able to learn more abstract algebraic rules such as “AAB” (a repetition of any two items followed by a third one) [8,9]. This capacity for abstract rule learning seems to be enhanced in humans and to rely on inferior prefrontal cortex (“Broca’s area”) [10,11]. Furthermore, different but neighboring sectors of inferior prefrontal cortex appear to be used for linguistic and for mathematical rules [12,13]. The question therefore arises whether a capacity for the internal representation and manipulation of nested sequences also underlies the acquisition of mathematics. While there have been several studies of artificial language learning ([3,9,14–16]; see [17] for a review), there have been comparatively fewer studies of the acquisition of mathematical structures. Our aim here is to introduce a novel experimental paradigm to study the acquisition of elementary structures in the domain of geometry, with the ultimate goal of probing whether this ability presents some features that are uniquely developed in the human species (for a similar approach, see [18,19]).

Several recent studies have suggested that even uneducated humans possess proto-mathematical intuitions of geometry. Indeed, human abilities to navigate the environment and to recognize geometrical shapes appear to develop early [20,21], are shared with many different animal species [22–24], and rely on a precocious knowledge of geometrical notions like distance, direction, length, or angle [25,26]. Even adults who lack school education and whose language has an impoverished vocabulary for geometry, rely on abstract geometrical cues when processing shapes and maps [27,28]. In analogy with the domain of numbers [29,30], it seems reasonable to hypothesize that these basic geometrical intuitions may serve as foundation for more abstract ideas [31–33]. However, the mechanisms that lead to the formation of advanced mathematical concepts from simpler ones still remain unknown.

In the present paper, we propose to formalize the human sensitivity to mathematical rules as the availability of a “language of thought” [34] that allows the formation of complex representations from a small repertoire of primitives. Following Fodor’s ideas, such a language should comprise a limited set of atomic elements (“lexicon”) that can be combined into more complex representations thanks to a set of formal combinatorial rules [34–36]. Such an approach has already proved relevant to model human conceptual learning [37–39]. In the specific case of spatial learning, Yildirim et al. [40] introduced a compositional language for spatial sequences, including a cursor, a set of basic commands to move it, “goto” loops, and recursion. They show that this language could capture the behavior of human adults in categorizing auditory or visual spatio-temporal sequences drawn out of seven locations arranged around a circle. Yildirim et al. showed that their language could account for the transfer of abstract sequence knowledge from the visual to the auditory modality (and vice-versa). However, their language did not model the participants’ understanding of geometry. Geometrical primitives such as symmetry were unnecessary for their purposes, since the spatial sequences were drawn from 7 locations on the circle and therefore did not form regular geometrical shapes (unlike the present work). Only a handful of researchers have explicitly focused on geometrical learning. Coding languages such as LOGO, a language in which a child learns to give directional instructions to a turtle walking across a page, have been used to produce regular geometrical patterns [41]. Following Chomsky’s ideas, Leyton introduced a generative grammar that partially captures the human perception of geometrically regular static shapes [42,43]. These research programs, however, either lacked empirical testing or were designed for educational purposes, and they did not systematically probe the human acquisition of geometrical sequences.

Lying at the intersection of those previous efforts, the present work introduces a simple formal language composed of geometrical primitives and combinatorial rules that suffice to describe the symmetries of a regular octagon. We ask whether humans can use such primitives and combine them in order to encode regularities of variable degree of complexity in spatial sequences. By analyzing the speed and ease with which human adults and children detect and memorize geometrical structures, we show that our language provides an adequate description of the representation that humans use to encode spatial sequences. By testing their capacity to anticipate the rest of the sequence, even before it has been fully presented, we examine how quickly human adults and children learn such combinatorial rules. By testing a variety of sequences, we probed the complexity of the rules that can be acquired. Using these data, we outline a theory of rule complexity consistent with human behavior.

### Language

**Fig 1:**
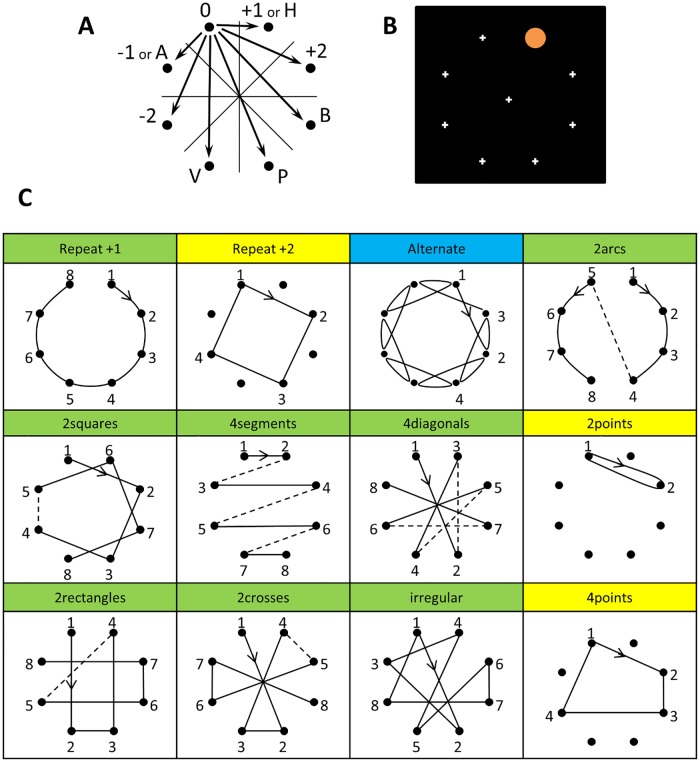
Paradigm. (A) Basic geometrical rules used to create sequences: rotations (+1, +2, -1, -2), axial symmetries (H: horizontal, V: vertical, A,B: oblique) and rotational symmetry (P). From one location of the octagon, each of the 7 others can be reached by the application of one or more primitives. (B) Screen shot from experiment 1. The orange dot appears at successive locations on the octagon, and subjects are asked to predict the next location. (C) Examples of sequences presented to French adults (blue), kids and Munduruku adults (yellow), or both (green).

We designed a formal language capable of describing, in a compact manner, all sequences of movements on a regular octagon. The set of primitive instructions is shown in Fig 1A and includes rotations, axial and point symmetries. Each of these instructions captures a possible transition from one location on the octagon to another. We denote them as 0 (stay at the same location), +1 (next element clockwise), +2 (second element clockwise), -1, -2, H (horizontal symmetry), V (vertical symmetry), P (rotational symmetry, equivalent to +4), A and B (symmetries around diagonal axes).

From these primitives, a sequence can then be generated by simple concatenation (e.g. the expression +2 +2 +2 +2 generates a square). Although any sequence can be encoded in this manner, we will provide evidence that humans detect and encode regular sequences in a much more compressed form. Thus, we also assume that the “language of thought” includes instructions for repeating operations. For instance, the sequence +2 +2 +2 +2 may be encoded as [+2]^4, i.e. four repetitions of +2). The language also allows for a more complex form of “repetition with variation”, as when drawing a first square, and then a second one rotated by one dot: the corresponding expression is denoted [[+2]^4]^2<+1>, where [+2]^4 encodes the square and []^2<+1> repeats it twice with an offset of +1 in the starting point. S1 Text presents a formal syntax and semantics of this minimal language for geometry.

In most languages, many equivalent expressions provide the same output. Here, for instance, the same square can be captured as +2 +2 +2 +2, [+2]^4, [+2]^3 +2, etc. We therefore assume that subjects apply Occam’s razor and attempt to select the most parsimonious expression that accounts for the observed sequence. The concept of Kolmogorov complexity, a notion from algorithmic information theory, provides a natural mathematical framework for these ideas [36,44]. This framework defines the complexity of a given sequence as the length of the shortest expression capable of producing it in a Turing-complete language, (i.e. any reasonable programing language).

Unfortunately, a classic result in algorithmic information theory is that, for any Turing-complete language, Kolmogorov complexity is not computable. Even for simple languages, Kolmogorov complexity is often difficult to compute in practice, because it involves examining, for each sequence, all the programs that compute it, a search that typically grows exponentially with the size of the sequence. Different methods have been developed to approximate Kolmogorov complexity. One idea is to approximate it using standard file compressors such as Lempel-Ziv. Such approach was used e.g. in [45] to cluster large documents via a definition of universal distance. File compressors behave well in relatively large texts but fail to provide any significant compression when the input is a very small text devoid of repetitions, such as the spatial sequences of 8 locations that we used here. In our case, we thus defined a new language capable of detecting specific geometrical patterns in such short sequences. To quantify sequence complexity, we used the notion of “minimal description length” which is closely related to Kolmogorov complexity [46] (for other uses of information theory and minimal description length in psychology, see e.g. [38,47–52]). From now on, we call “complexity” of a spatial sequence x, denoted K(x), the length of the shortest expression(s) in our language that reproduces it. The corresponding psychological assumption, that we put to a test in our experiments, is that human participants attempt to “compress” the spatial sequence mentally, i.e. to minimize the memory cost by identifying the simplest (shortest) mental expression that allows them to store the sequence.

We make the simplest possible assumptions regarding expression length (see S1 Text for details). In essence, (1) each additional primitive instruction adds a fixed cost; (2) repeating a set of instructions *n* times adds a cost proportional to log(n) to the instructions to be repeated; (3) the relative size of those two costs is such that even a single repetition reduces the size of an expression (thus, the expression“[+2]^2” is more compressed than the equivalent “+2 +2”).

### Stimulus sequences

In all experiments below, our general aim was to (1) probe human memory for spatial sequences on the octagon and (2) examine whether human behavior could be captured by our formal language and our definition of complexity. To this aim, we first generated all the 5040 sequences of length 8 that could be generated on the octagon, beginning in the same origin and without repetition of any specific location. We then computed their complexity (K) in the above language, quantifying their degree of geometrical regularity. Finally, we selected sequences that spanned a broad range of geometrical primitives and regularities. All sequences used in experiments 1–4 are shown in Fig 1C. We now detail them:

- The most complex sequences (K = 16), called “irregular”, consisted in a serial presentation of all 8 locations in a fixed order with no apparent regularity. Such sequences could also be called “incompressible” because their minimal description consists in a mere list of successive transitions between locations, without any compression afforded by repetition. Our language comprised 768 such maximal-complexity sequences. For any given subject, one of them was chosen randomly. In order to probe sequence memory, it was then repeated a second time, for a total of 16 locations.
- At the other extreme, the sequence called “repeat” (K = 5) contained a single repeated primitive (either +1 or -1), and thus consisted in a simple clockwise or counterclockwise progression.
- The “alternate” sequence (K = 7) was constructed by applying alternatively two steps in one direction (either +2 or -2), and one step in the opposite direction (respectively -1 or +1). Thus, this sequence involved no nesting, but a mere repetition of two instructions.

Other sequences contained two embedded levels of regularity: a lower level where instructions built a geometrical shape (e.g. a square), and a higher level at which the shape was repeated with a global transformation (e.g. the square was rotated):

- The “2squares” sequence (K = 8) was constructed by applying three times the rule +2, thus drawing a square, and then restarting with a rotated starting point, which was defined by applying the rule +1 or -1 to the previous starting point.
- The “2arcs” sequence (K = 8), consisted in three applications of the rule +1 (thus drawing an arc of four successive points), then globally flipping this figure using an axial symmetry in order to complete it with the four remaining locations.
- The “4segments” sequence (K = 7) consisted in first drawing a segment by applying an axial symmetry, then translating it four times by shifting its starting point. This sequence resulted in a succession of four parallel segments connected by a zigzag shape.
- The “4diagonals” sequence (K = 7) was constructed similarly through the repeated application of rotational symmetry to four consecutive starting points.

Finally, two sequences contained three embedded levels of regularity.

- The “2rectangles” sequence (K = 10) consisted in an initial segment on which a global axial symmetry was applied (thus tracing a rectangle), and then a +2 rotation that transposed this shape to the remaining four points.
- The “2crosses” sequence (K = 7), similarly, started with a rotational symmetry (diagonal segment), which was then transformed by an axial symmetry (thus tracing a cross), and then a +2 rotation that transposed it to the remaining four points.

In experiments 2–4, to evaluate memory span, we added two sequences that spanned only a subset of the 8 locations. These were irregular sequences with respectively 2 and 4 locations (called “2points” [K = 6] and “4points” [K = 9]).

## Results

### Experiment 1

#### Ethics statement

Experiments were approved by the regional ethical committee (Comité de Protection des Personnes, Hôpital de Bicêtre), and participants gave informed consent.

#### Participants

Participants were 23 French adults (12 female, mean age = 26.6, age range = 20–46) with college-level education.

#### Procedure

The experiment was organized in short blocks. In each block, subjects were presented with a specific sequence of spatial locations, which they were asked to continue. The eight possible locations, forming a symmetrical octagon, were constantly visible on the computer screen (Fig 1B). On a given trial, the locations forming the beginning of the chosen sequence were flashed sequentially, and then the sequence stopped. The subject’s task was to guess the next location by clicking on it. As long as the subject clicked on the correct location, he was asked to continue with the next one. In case of an error, the sequence was restarted from the beginning: the entire sequence of locations was flashed again, the mistake was corrected, and the subject was again asked to predict the next location. For each sequence, the procedure was initiated by showing only the first two items. Thus, starting with the 3^rd^ location in the sequence, subjects were given a single opportunity to venture a guess at each step. In order to introduce the task, participants were always presented first with a “repeat” sequence of clockwise or counterclockwise rotating locations. The order of subsequent sequences was randomized.

#### Stimuli

On each block, a spatial sequence consisting in a succession of 16 locations was presented. These sequences are shown in blue and green labels in Fig 1C. In total, each participant was presented with two “repeat”, two “alternate” and two “2squares”, each spanning the two directions of rotation around the octagon. Two “2arcs”, four “4segments” and one “4diagonals” were also presented in order to test the comprehension of all four axial symmetries and rotational symmetry. In these cases, the direction of rotation was randomized. One exemplar of “2rectangles” and one of “2crosses” were also randomly selected. Finally, two irregular sequences were picked randomly among the 768 sequences of maximal complexity. The starting point of each sequence was picked randomly among the subset of eight locations of the octagon that preserved the global shape.

#### Statistical analysis

The data consisted in a discrete measure of performance (correct or error) for each subject, each sequence item, and each ordinal position from 3^rd^ to 16^th^. Because those data were discrete (even after averaging performance over a subset of sequences or ordinal data points), we used Friedman’s non-parametric test for paired data (a non-parametric test similar to a parametric repeated-measures ANOVA). When necessary, we used a Bonferroni correction for multiple comparisons (across 14 data points for educated adults, 8 data points for other subjects). To quantify the evolution of performance over time, we calculated for each subject the Spearman’s rank correlation of error rates with ordinal position, and compared the mean correlation coefficient to 0 using a Student t-test. When the evolution of performance over time was evaluated on a small number of ordinal positions (3 or 5, as happens in experiments 2–4), we used Friedman’s test for multiple conditions. Finally, whenever we needed to compare performance between groups of subjects on a specific condition (e.g. adults and children, as will arise in experiment 2), given that we had discrete measures (correct or error), we used Fisher’s exact test when the number of measures per subject was 1 or 2; and the Wilcoxon rank-sum test for independent samples whenever comparing the means of 3 or more conditions.

Specific planned comparisons were performed in order to finely probe the understanding of hierarchical sequence structure. For example, in “4segments”, the even data points correspond to the application of the 1^st^-level, shallower level of regularity (axial symmetry), while the odd data points result from a change of starting point, and thus represent a deeper, 2^nd^-level regularity that involves a non-adjacent temporal dependency (subjects must remember the starting point of a sub-sequence of 2 items). Consequently, comparing performance on such data points provides information about the representation of nested rules in our paradigm.

#### Results

**Fig 2:**
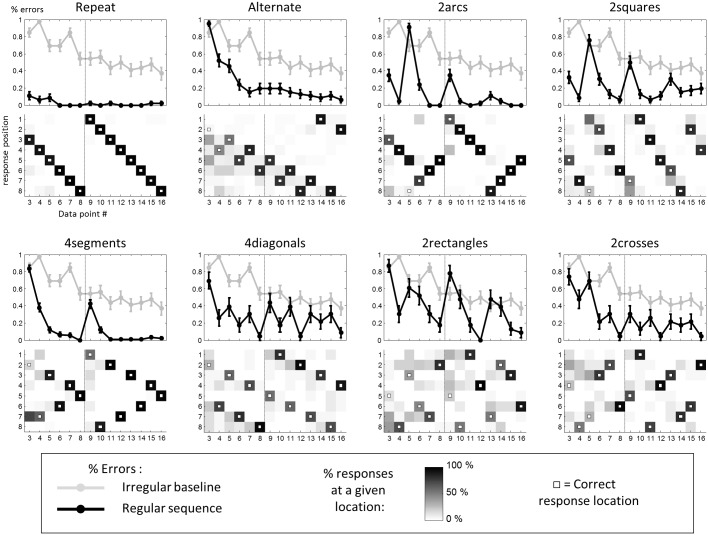
Performance of adult participants in experiment 1. Top panels show the evolution of error rate across successive steps (data points 3–16 in adults) for each regular sequence (error bars = 1 SEM). The gray curve in the background shows the error rate for irregular sequences, which serve as a baseline. Bottom panels show the percentage of responses at a given location for each data point. White dots indicate the correct location. Vertical dashed lines mark the transition between the two 8-item subsequences that constitute the full 16-item sequences.

As a baseline, we first examined the performance with “irregular” 8-item sequences, which contained no obvious geometrical regularity. The evolution of average performance across the two successive repetitions is shown as a background gray curve in all panels of Fig 2. The mean error rate decreased across trials (mean rank correlation of error rate with ordinal position: ρ = -0.51 ± 0.05, Student t-test: t_22_ = 10.3, p <7.10^−10^). This improvement could be decomposed into two contributions: rote memory and anticipation. First, performance was better in the second half of each block, i.e. during the repetition of the sequence, than in the first half, when the sequence was introduced, indicating rote memory (Friedman test: F = 15.7, p<10^−4^; point-by-point comparisons revealed a significant difference at all but the last location, p*s*<0.05). Second, performance improved even within the first half, even before the full sequence had been presented (anticipation; r = -0.4 ± 0.08, Student t-test: t_22_ = 5.1, p <4.10^−5^). This finding indicates that subjects took advantage of the fact that the 8 locations were sampled without replacement, thus narrowing the choice of remaining locations. Yet memory for past locations was not perfect, as shown by the fact that performance on data points 7 and 8 remained worse than the chance level expected if subjects perfectly avoided past locations (respectively 85 ± 6% vs 50%; and 54 ± 8% vs 0% errors; One-Sample Wilcoxon Signed Rank Tests: both p*s*< 0.001).

Irregular sequences served as a baseline with which to compare other regular sequences. In every regular sequence, the mean error rate was significantly lower than in the irregular baseline (“repeat”: 2.5 ± 0.9%; “alternate”: 25.5 ± 4%; “2arcs”: 15 ± 1.4%; “2squares”: 23.5 ± 3.7%; “4segments”: 15 ± 1.4%; “4diagonals”: 27 ± 4%; “2rectangles”: 38 ± 3.2%; “2crosses”: 27.5 ± 3.2%; “irregular”: 59.5 ± 3.8%; Friedman tests, all p*s*<0.001). Moreover, in every case, participants performed significantly better than baseline even before the full presentation of the 8-item sequence (averaged error rate of data points 6–8 for “repeat”: 0%; “alternate”: 19.6 ± 6.1%; “2arcs”: 8 ± 2.1%; “2squares”: 16.7 ± 5%; “4segments”: 4 ± 2%; “4diagonals”: 17.4 ± 4.7%; “2rectangles”: 33.3 ± 5.2%; “2crosses”: 18.8 ± 5.2; and “irregular”: 69.6 ± 3.8%; all p*s*<10^−4^).

**Fig 3:**
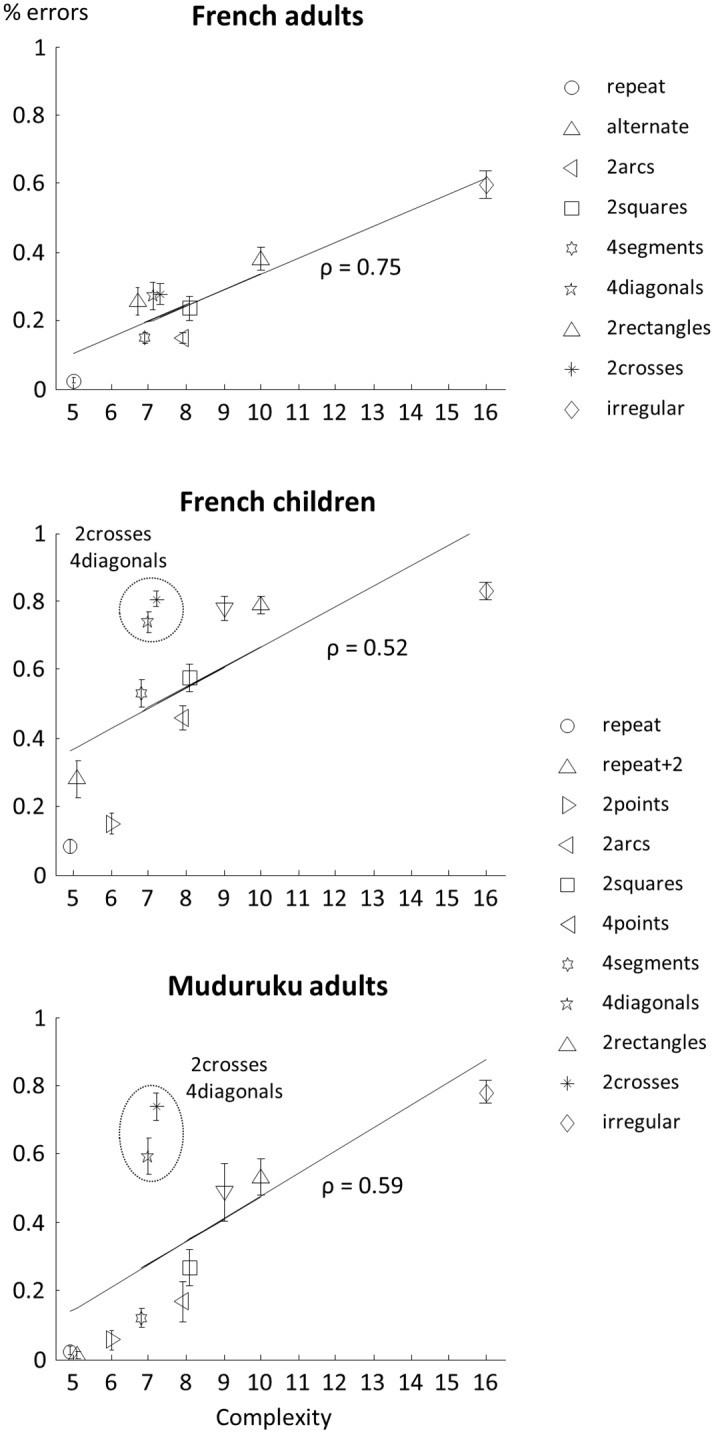
Complexity predicts error rates. For each sequence, the y axis represents the mean error rate, and the x axis the sequence complexity, as measured by minimal description length. Panels show data from French adults (top, experiment 1), preschool children (middle, pooling over experiments 2 and 3), and Munduruku teenagers and adults (bottom, experiment 4). For each group, a regression line is also plotted and the Spearman’s correlation coefficient is displayed. In French children and Munduruku adults, the “4diagonals” and “2crosses” are clear outliers—as explained in the main text, the regression can be improved by assuming that their “language of thought” does not include rotational symmetry P.

Thus, sequence regularity facilitated both rote memory and anticipation. Crucially, as predicted, these effects were captured by our measure of complexity: the mean error rate was highly correlated with K across sequences (for all data points: Spearman’s ρ = 0.75 ± 0.04, Student t-test: t_22_ = 21, p <10^−11^; for data points 6–8: ρ = 0.73 ± 0.04, t_22_ = 21, p < 10^−9^, Fig 3, top panel). Furthermore, complexity in our language gave a better account of adults’ behavior than alternative encoding strategies which did not use geometrical features such as rotations and symmetries, but used only the distance between successive locations. We computed two variants of sequence complexity devoid of geometrical content: the normalized jump length, measuring the average distance between locations in a sequence, averaged over the number of jumps; and complexity in a degraded language where the primitives were only ±1, ±2, ±3, +4, and repetition (S1 Fig). In both cases, obvious outliers were observed (e.g. the complexity for “4segments” in the second case reached the maximum value of 16, which is inconsistent with the data). Moreover, correlations of those measures with total error rate were significantly lower than those obtained with the full language (normalized jump length ρ = 0.60 ± 0.03, t(44) = 3.23, p = 0.003; complexity in degraded language: ρ = 0.51 ± 0.03, t(44) = 4.88, p < 10^−4^).

We then examined the pattern of errors in each regular sequence. Unsurprisingly, for the “repeat” sequence, which only consisted in the repeated application of the +1 or -1 rule, all error rates verged on 0 and were far below the baseline (all p*s*< 0.001 corrected). The fact that subjects were already able to complete the sequence after seeing only the first two items suggests that they quickly recognized and applied the primitives+1 and -1, and treated repetition as a default assumption.

For “alternate”, after a systematic error at the 3^rd^ data point (error rate = 95%), the error rate continuously decreased over the first half of the sequence (mean correlation coefficient: ρ = -0.68 ± 0.06, Student t-test: t = 11.8, p < 5.10^−11^) and dropped to 15 ± 6% at the 7^th^ data point. Even though “alternate” induced more errors than “repeat” (overall: F = 23, p< 10^−6^), performance was significantly better than “irregular” (all p*s*< 0.05 corrected, except at the 3^rd^ and the 5^th^ data points). Thus, although “alternate” was more difficult than “repeat”, participants were able to identify and combine the rules +1 and +2.

For“2arcs” and “2squares”, performance profiles were similar. At all data points except the 5^th^, 9^th^, 13^th^ and 16^th^, error rates were significantly below the baseline (all p*s*< 0.05 corrected). The data points with high performance correspond to the application of the lowest-level rule (+1 for “2arcs” and +2 for “2squares”), therefore providing evidence that this superficial rule was quickly learned. On the contrary, data points 5, 9 and 13, corresponding to the application of the higher-level rule, exhibited more errors than their neighbors (Friedman test: F = 23, p = 2.10^−6^). At data point 5, the error rate was not significantly below the irregular baseline in “2squares”, and it was even worse than baseline in “2arcs” (error rate at 5^th^ data point in “irregular”: 70 ± 6%; “2arcs”: 91 ± 4%, F = 6.23, p = 0.013; “2squares”: 76 ± 8%, F = 0.69, p = 0.41). Errors at this point consisted primarily in the continued application of the lower-level rule. Importantly, however, performance on data point 5, 9 and 13 improved over time (“2arcs”: Friedman test: F = 37, p < 9.10^−9^; “2squares”: F = 18.6, p < 9.10^−5^), and error rates at data points 13 fell significantly below baseline in “2arcs” (p< 0.05 corrected), indicating that subjects eventually learned both 1^st^ and 2^nd^-level rules.

For”4segments”, error rate fell significantly below baseline for all data points (all p*s* < 0.001 corrected), except points 3 and 9. Within each block of 8 items, error rate decreased quickly and continuously to 0 (rank correlations for the 1^st^ half: ρ = -0.82 ± 0.02, t_22_ = 36.4, p < 0.001; and the 2^nd^ half: ρ = -0.62 ± 0.04, t_22_ = 15.8, p = 2.10^−13^). These results suggest that the 1^st^ and 2^nd^-level rules forming the “4segments” sequence were easily identified and applied. Separate analyses indicated that the mean error rate was similar for horizontal, vertical, and oblique symmetries (vertical: 11.5 ± 1.6%; horizontal: 16.1 ± 2.8%; oblique: 16.8 ± 2.1% and 15.5 ± 2.5%; Friedman test for differences between the four types of symmetries: F = 4.3, n.s.). Thus, adult participants easily identified all axial symmetries.

The performance in“4diagonals” indicated that rotational symmetry was harder to identify than other symmetries (comparison of “4diagonals” and “4segments”; respectively 27.3 ± 4% vs 15 ± 1.4% errors, F = 7.3, p = 0.007). A saw tooth pattern (Fig 2) indicated that even data points had systematically lower error rates than odd ones (Friedman test: F = 18, p < 3.10^−5^), suggesting that the application of rotational symmetry (1^st^-level rule) was easier than that of the rotation of the starting point (2^nd^-level rule). Even data points exhibited error rates significantly lower than baseline (all p*s*< 0.02, p*s* < 0.001 corrected except for data points 10, 14 and 16). On the contrary, odd data points exhibited no difference with baseline, again suggesting that the 2^nd^-level rule was harder to understand than the 1^st^-level one. Nevertheless, there was a small but significant improvement over time on both odd and even data points (rank correlation for odd data points: ρ = -0.4 ± 0.07, t = 5.5, p < 2.10^−5^; rank correlation for even data points: ρ = -0.39 ± 0.06, t = 7.32, p < 6.10^−11^).

In “2 rectangles”, like in “2squares”, data points 5, 9 and 13 corresponded to the application of the deepest (3^rd^-level) rule. None of these exhibited an error rate lower than the baseline (data point 5: 60.9 ± 10.6% vs 69.6 ± 6.2%, F = 0.28, p = 0.6; data point 9: 78.2 ± 9% vs 54.3 ± 8.5%, F = 4, p = 0.046; data point 13:47.8 ± 10.9% vs 41.3 ± 9.5%, F = 0.69, p = 0.4), and there was no improvement over time (Friedman test: F = 4.1, p = 0.13), suggesting that participants did not manage to understand how the starting point of the rectangle changed. At the immediately subsequent data points 6, 10 and 14, that corresponded to the construction of the first side of the rectangle, performance improved compared to points 5, 9 and 13 (respectively 46 ± 7%vs 62 ± 5% errors, F = 2.88, p = 0.089), although it was still not significantly lower than baseline (F*s*< 0.5, p*s*> 0.4). At subsequent points (7, 8, 11, 12, and 15, 16), the error rate further improved (14 ± 4% errors, Friedman comparison with 3^rd^-level rule: F = 22, p < 3.10^−6^) and became significantly lower than baseline (all p*s*< 0.05 corrected), indicating that the 1^st^ and 2^nd^-level rules that allowed to complete the rectangle were systematically learned.

Finally, for “2crosses”, the performance profile resembled that of “4diagonals”: on even data points, the error rate was systematically lower than the baseline (all p*s*< 0.03 corrected except at the 14^th^ data point) and globally lower than the error rate on odd data points (F = 10.7, p = 0.001), indicating that participants easily identified the most superficial rule. Additional evidence for a 3-tiered organization was observed. The error rate was significantly higher on data points 5, 9 and 13, corresponding to the starting point of the cross (3^rd^-level rule, 41 ± 7% errors) than on data points 7, 11 and 15, corresponding to the starting point of the second branch of the cross (2^nd^-level rule 26.1 ± 7% errors, Friedman comparison between 2^nd^ and 3^rd^ levels: F = 4.45, p = 0.035). No such difference was seen between data points 5, 9, 13 and 7, 11, 15 in “4diagonals” (F = 1.9, p = 0.17). On data point 7, 11 and 15, the error rate was in turn significantly higher than on subsequent data points 8, 12 and 16, corresponding to the completion of the cross (1^st^-level rule, 4.35 ± 3.3% errors, Friedman comparison between 1^st^ and 2^nd^ levels: F = 5.33, p = 0.021). On data points 6, 10 and 14, corresponding to the construction of the first branch of the cross (17.4 ± 5.2% errors, the error rate was also significantly lower than on data points 5, 9 and 13 (F = 9.3, p = 0.002). Finally, on data points 3, 5, 11 and 15, the error rate was not significantly lower than the baseline. In summary, 2^nd^ and 3^rd^ levels rules, though eventually learnt, were harder to grasp than the 1^st^ level rule.

#### Discussion

Adults were able to detect various geometrical regularities and to quickly generalize on the basis of only a few items, before seeing the entire sequence. They correctly prolonged every sequence and erred precisely at the points where past clues did not allow them to guess the requested rule (data point 3 in “alternate”, “4segments”, “4diagonals”, “2rectangles” and “2crosses”; data point 5 in “2arcs”, “2squares”, “2rectangles” and “2crosses”, and data point 9 in “4segments”). In most such cases, systematic errors indicated that subjects systematically continued to apply the lower-level rule. For example, in “2squares”, participants got used to a succession of +2 rules and kept applying it at the 5^th^ data point. In other cases where the previous points formed a sub-sequence that seemed to come to an end (e.g. after the first “4 points” in“2rectangles” and “2crosses”, or after the first 8 points in “4segments”), participants failed because they could not guess how to restart.

Aside from these predicable errors, our results indicated that all regular sequences were better learnt than the irregular baseline, with error rates increasing essentially monotonically with complexity. This finding indicates that geometrical regularity is a major determinant of visuo-spatial memory in our task. Indeed, geometrical regularities allowed participants to memorize sequences of 8 items and beyond that would have otherwise exceeded their working memory capacity (as exemplified by the persistence of errors in the “irregular” baseline).

Participants’ performance provided clear indications of the type of regularities that they were able to identify. All the primitives that we hypothesized were easily recognized by adult subjects: +1/-1 (successor), +2/-2, and all axial and point symmetries (as indicated by superior performance on even data points of “4segments” and “4diagonals” sequences). Furthermore, participants also identified additional embedded levels of regularity. Performance with “2arcs”, “2squares”, “4segments” and “4diagonals” sequences provided evidence for a fast learning of the most superficial rule and its repetition. 2^nd^ and 3^rd^-level rules were harder to learn, as suggested by (1) the slower decrease of error rates for 2nd level than for 1st level, and (2) the persistence of errors over time at data points corresponding to the 3^rd^-level rule in “4diagonals”, “2rectangles”, “2crosses”. By construction, evidence in support of those deeper levels is presented with reduced frequency compared to the 1^st^-level rule—for instance in “2arcs” and “2squares”, the 2^nd^-level rule applies only to one trial in four. However, sequences such as “4diagonals” and “2crosses”, where 1^st^- and 2^nd^-level rules apply with the same frequency (every other trial), the 2^nd^-level rule still induced more errors than the 1^st^-level rule. Those results therefore suggest that deeper hierarchical levels are genuinely harder to learn, probably because they involve non-adjacent temporal dependencies: in “2arcs” or “2squares”, for instance, the 2^nd^-level rule applies to the initial point of a length-4 sub-sequence. Another compounding factor may be spatial distance across space. The “4diagonals” or “2crosses”, in which the distance between odd locations is almost maximum, yielded the maximum error rates.

Altogether, these findings indicated that adult participants easily identified elementary primitives of symmetry and rotation, and promptly understood the hierarchical organization of regular sequences. However, such performance is perhaps unsurprising giving that our subjects were young adults with college-level education. In experiment 2, we asked whether preschoolers, who have not yet received formal education, also grasped geometrical rules.

### Experiment 2

#### Participants

24 preschoolers were tested (minimal age = 5.33, max = 6.29, mean = 5.83 ± 0.05). The experimental apparatus was installed at school, in a quiet room that was not the usual classroom. Children came one by one to play the game.

#### Procedure

To render the experiment more attractive for young children, we replaced the flashing dots with pictures of animals, one for each sequence. Children were asked to look carefully at how each animal moved. They were told that animals were playful: they appeared at one place, and then hid at another. Children were asked to catch them by pointing at the next location where they thought that they might appear. The experimenter then clicked on the designated target. To shorten the experiment, we divided each trial into two subsequences of 8 items. Children saw the first five locations of a sequence and had to point to the next three. Then, after a short break, they saw the first three locations of the same sequence and had to point to the next five. Like in adults’ experiment, whenever kids pointed to the wrong location, the program automatically restarted from the beginning of the trial, went on to correct the error, and asked for a guess of the next location.

#### Stimuli

The sequences were essentially the same as in experiment 1 (yellow and green labels in Fig 1C). Only the sequence “alternate”, which was difficult even for adults, was replaced by a sequence that allowed us to test directly for kids’ understanding of the basic rule +2. This sequence consisted in the successive application of the rule +2 (called “repeat+2”). To explicitly measure working memory span, we also introduced two additional baselines, i.e. irregular sequences with only 4 and 2 locations (called “4points” and “2points”). Finally, to reduce the duration of the experiment, we presented only a single exemplar of each sequence category. The only exception was the“4segments”sequence, which was presented 4 times in order to test all 4 axial symmetries.

#### Results

We first analyzed performance on the “irregular” baselines with 8, 4 and 2 items. When 8 locations devoid of any geometrical regularity were presented, the error rate was very high (80 ± 2% errors in average). Yet notably, as for adults, the performance improved over time (Spearman’s rank correlation over the two presentations: ρ = -0.41 ± 0.04, Student t-test: t_23_ = 10.4, p < 4.10^−10^). Surprisingly, no such a pattern of error was observed for “4points”in which the error rate remained at a sustained level during the whole trial (minimum error rate: 75 ± 9%). There was no significant improvement neither in the first presentation phase, nor in the second (Friedman’s test on 1^st^ and 2^nd^ phases: F*s* = 0.29; 3.2; p*s*> 0.5). However, error rates for “2points” significantly differed from “irregular” (from data points 7 to 16, all p*s* < 0.01 corrected) and significantly decreased over the first phase (F = 19.7, p = 10^−4^). Thus, measured with our method, children’s visual memory span for irregular sequences fell between 2 and 4.

For most of the regular sequences, the mean error rate was significantly lower than the “irregular” baseline (Friedman’s tests: all p*s*<0.002 either across 1^st^ and 2^nd^ phases or for 1^st^ phase only): “repeat” (across 1^st^ and 2^nd^ phases: 6 ± 2% errors; on 1^st^phase only: 13 ± 5%), “repeat+2” (1^st^ and 2^nd^ phases: 24 ± 7%; 1^st^ phase only: 33 ± 9%), “2arcs” (39 ± 5%; 49 ± 6%), “2squares” (53 ± 6%; 51 ± 8%) and “4segments” (50 ± 5%; 54 ± 5%). However, such a performance was not seen for “4diagonals” (73 ± 4% errors), “2rectangles” (79 ± 3% errors), “2crosses” (81 ± 3% errors), for which mean error rates did not differ from baseline (all p*s*> 0.07).

As with adults, we found that preschoolers’ overall mean error rate was predicted by the complexity of the sequences (at all data points: Spearman’s ρ = 0.52 ± 0.02, Student t-test: t_23_ = 21, p <10^−9^; at data points 6–8: ρ = 0.41 ± 0.03, t_23_ = 11, p < 10^−5^, Fig 3, middle panel), even though the correlation was lower than in experiment 1 (t_46_ = 5, p < 10^−5^).

**Fig 4:**
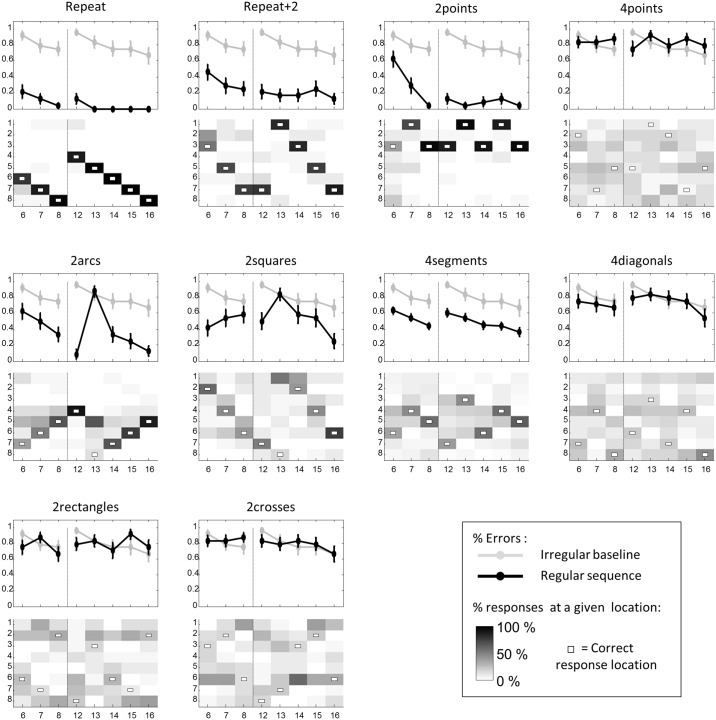
Performance of preschool children in experiment 2. Same format as Fig 2. In children, only data points 6 to 8 and 12 to 16 were collected. Vertical dashed lines indicate the transition between the first and the second presentations of the 8-item sequences.

Examination of individual sequences shown in Fig 4 revealed that, for “repeat”, all error rates dropped quickly to 0 and were far below the baseline (all p*s*< 8.10^−4^ corrected), indicating that children quickly recognized and applied the primitives +1 and -1. The same conclusion was reached for the primitives +2 and -2 in “repeat+2”, in which all error rates were significantly lower than baseline (Friedman test: all p*s*< 0.05 corrected), continuously decreased over the 1^st^ phase (F = 8.4, p = 0.15) and stayed close to 0 over the 2^nd^ phase.

As for adults, performance profiles were similar for “2arcs” and “2squares”. Error rates were below baseline at most of the data points (“2arcs”: all p*s*< 0.05 corrected except at data points 6, 7 and 13; “2squares”: p*s*< 0.05 corrected at data points 6, 12 and 16). These results therefore provide evidence that the superficial rule (+1 for “2arcs” and +2 for “2squares”) was quickly learned, while the application of the higher-level rule, at the 13^th^ data point, induced more errors (Friedman test of comparison between the 13^th^ data point its neighbors: F = 23, p = 2.10^−6^). At this particular data point, 67% of children simply continued to apply the 1^st^-level rule in “2arcs” and 54% in “2squares”.

For “4segments”, error rate was significantly below the baseline at almost all data points (Friedman test: all p*s*< 0.05 corrected at data points 6, 7, 12, 13 and 15) and decreased continuously within each presentation phase (1^st^ phase: F = 12.4, p = 0.002; 2^nd^phase: F = 11.9, p < 0.02). Separate analyses indicated that the mean error rate was similar for horizontal, vertical, and oblique symmetries (vertical: 46 ± 7% errors; horizontal: 42 ± 6%; oblique: 55 ± 7% and 58 ± 6%; Friedman test for differences between the four types of symmetries: F = 4.9, n.s.). Thus, all axial symmetries forming the 1^st^ level of the “4segments” sequences were correctly identified and applied. Moreover, at odd data points of “4segments”, which correspond to the application of the 2^nd^-level rule, performance was significantly better than baseline (all p*s*< 0.05 corrected), therefore indicating that children also discovered the 2^nd^-level rules.

For “4diagonals”, error rate was not significantly below baseline neither at even data points, corresponding to the application of the 1^st^-level rule, i.e. rotational symmetry, nor at odd data points, corresponding to the application of the 2^nd^-level rule (all p*s*> 0.1). This result suggests that rotational symmetry was more challenging than axial symmetries for 5-years-old children.

Finally, for “2rectangles” and “2crosses” that contain 3 embedded levels of rules, none of the data points showed an error rate significantly lower than the baseline (all p*s*> 0.1). These rules seemed to be beyond the grasp of our children.

#### Discussion

Kids experienced more difficulty than adults, but their answers still provided evidence for a quick understanding of most geometrical primitives: they mastered +1 and +2 operations as well as axial symmetries, and only failed with rotational symmetry. Their behavior with the category “4segments” demonstrated that they could detect embedded regularities, yet they failed with more complex embeddings that defined the changes in the starting point of arcs, squares, rectangles or crosses. It thus seems that a reduced language, with fewer primitives and shallower embeddings, is needed to capture children’s performance. In the final section, we will provide a formal model of this idea.

One possibility is that children failed to detect sequential dependencies that exceeded their spatial working memory span. Performance on the “4points” irregular sequence suggested that their spatial memory span was below 4, while the “2arcs”, “2squares”, “2rectangles” and “2crosses” sequences involved dependencies spanning over 4 locations. This limitation could also explain the errors children made in “4diagonals”: even if they partially understood what the regularity was, they remained confused about distant locations.

An alternative explanation for the children’s failures is the sequences were not repeated long enough. Indeed, the simplifications that we introduced implied that children were presented with fewer sequence repetitions than adults. This is because, when subjects failed, the entire sequence was repeated, and there was more opportunity for failing in the adults than in the children’s version of the experiment. For instance, when kids were asked to guess the 13^th^ location of a sequence, they had had at most 3 occasions to grasp the corresponding regularity on previous trials, while adults had up to 7 such occasions (assuming they frequently failed on previous trials). To address this issue, in experiment 3 we presented children with two complete previews of each sequence before the test phase started.

### Experiment 3

#### Participants

Participants were 23 5-years-old children (minimal age = 4.67, max = 5.85, mean = 5.41 ± 0.07), tested at school during school-day.

#### Stimuli and procedure

The experiment was identical to experiment 2, except that each block started with two full viewings of the corresponding 8-location sequence, while the child was merely instructed to attend carefully. This provided an opportunity to memorize the sequence before the testing phase began.

#### Results

**Fig 5:**
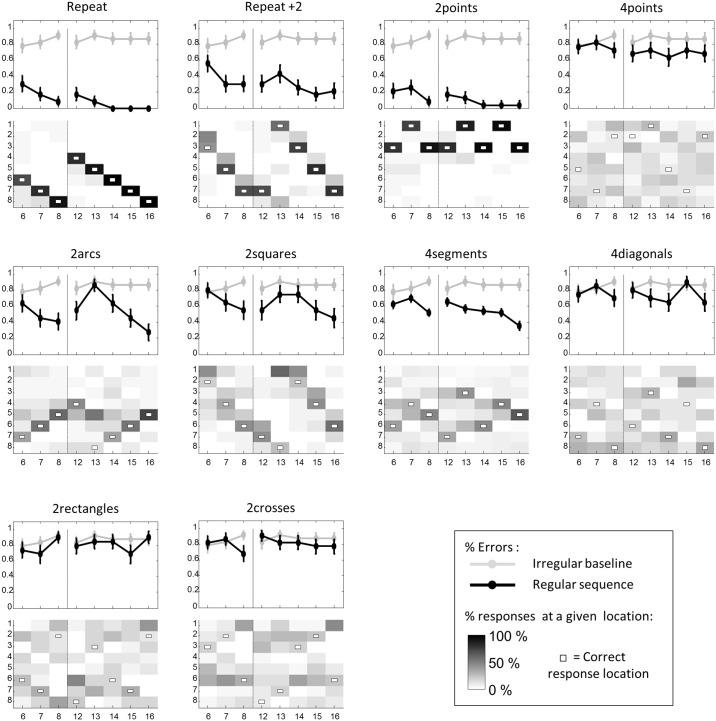
Performance of preschool children in experiment 3. Same format as Fig 4.

In spite of the additional training, the children’s results remained virtually unchanged (Fig 5). Comparisons of experiments 2 and 3, at each data point of each category, indeed revealed no significant improvement.

In details, the mean error rate remained very high for “irregular” (86 ± 3%)and “4points” (73 ± 6%) sequences, and there was no significant improvement of performance neither in the first phase, nor in the second phase (“irregular”: 1^st^ phase: F = 1.4, p = 0.5; 2^nd^ phase: F = 0.83, p = 0.9; “4points”: 1^st^ phase: F = 0.5, p = 0.78; 2^nd^ phase: F = 1.17, p = 0.88). In “2points”, mean error rate equaled 13 ± 4%, and at all data points, error rate significantly differed from “irregular” (all p*s* < 0.006 corrected).

Again, for “repeat”, “repeat+2”, “2arcs”, and “4segments”, the mean error rate was significantly lower than baseline (Friedman test: all p*s*< 0.007): “repeat” (across 1^st^ and 2^nd^ stage: 10 ± 3% errors; on 1^st^ stage only: 19 ± 6% errors), “repeat+2” (32 ± 8%; 39 ± 9% errors), “2arcs” (55 ± 6%; 52 ± 9% errors), and “4segments” (56 ± 6%; 61 ± 7% errors). In this experiment, the mean performance in “2squares” (overall error: 68 ± 6%; 1^st^ stage: 71 ± 8% errors) did not differ from baseline (F = 2, n.s). “4diagonals” (78 ± 4%; 80 ± 6% errors), “2rectangles” (83 ± 4%; 81 ± 6% errors), “2crosses” (82 ± 3%; 80 ± 5% errors), remained more challenging for children, with mean error rates not different from baseline (all p*s*> 0.15).

We again found a positive correlation between the mean error rate and the complexity of the sequences (at all data points: Spearman’s ρ = 0.52 ± 0.02, Student t-test: t_22_ = 19, p <10^−8^; at data points 6–8: ρ = 0.41 ± 0.04, t_22_ = 10, p < 10^−4^). Again, the correlation was weaker in children than in adults (t_45_ = 5, p < 2.10^−5^). Pooling across experiments 2 and 3, we found a global correlation between error rate and complexity equal to 0.51 ± 0.02 (t_46_ = 23, p < 10^−12^), again significantly weaker than in adults (t_69_ = 5.8, p < 10^−6^).

As in experiment 2, error rates on “repeat”, “repeat+2” and “4segments” were significantly better than baseline, and performance significantly improved over time, thus confirming that children were able to detect and use the primitive rules +1, +2 and axial symmetries (“repeat”: all p*s*< 0.007 corrected; improvement for 1^st^ and 2^nd^ stages: F*s* = 5.4; 11.6; p*s*< 0.05; “repeat+2”: all p*s*< 0.022 corrected except at the 6^th^ data point; improvement for 1^st^ and 2^nd^ stages: F*s* = 9; 11.2; p*s*< 0.03; “4segments”: all p*s*< 0.05 corrected except at data points 6, 7 and 12; improvement for 1^st^ and 2^nd^ stages: F*s* = 8.9; 15.6; p*s*< 0.02). As in experiment 2, children’ results on“4segments” were not influenced by the type of axial symmetry (vertical: 50 ± 8% errors; horizontal: 57 ± 7%; oblique: 66 ± 7% and 56 ± 8%; Friedman test for differences between the four types of symmetries: F = 2.5, n.s.). Error rate on “4diagonals” was not significantly better than baseline (all p*s*> 0.1), indicating that children again experienced more difficulty with rotational symmetry.

As in experiments 1 and 2, “2arcs” and “2squares” showed similar error patterns. “2arcs” provided evidence for the comprehension of the superficial rule: error rate was significantly below baseline at data points 8, 15 and 16 (p*s*< 0.05 corrected) and there was a significant improvement of performance over the 2^nd^ stage (F = 17.8, p < 0.002). For “2squares”, error rate was not significantly below baseline, but there was a tendency at data points 8, 15 and 16 (p*s*< 0.02 uncorrected). As in experiment 2, error rate at the 13^th^ data point of “2arcs” and “2squares” was at baseline level (p*s*> 0.2).

Finally, no evidence of learning was found in “2rectangles” and “2crosses”, for which error rate was not different from baseline (all p*s*> 0.2) and no performance improvement was observed (p*s*> 0.3).

#### Discussion

In spite of two additional viewings of the complete sequence, experiment 3 fully replicated experiment 2, thus affording several conclusions. First, +1, +2, and axial symmetries are geometrical primitives in children. Second, preschoolers are sensitive to embedded regularities in the “4segments” sequence. Third, under the present conditions, they fail to grasp more complex embedded regularities. Previewing the sequences did not influence performance, suggesting that the latter conclusion cannot be attributed to a lack of exposure to sufficient evidence.

The difficulties that 5-year-old children experienced with rotational symmetry and with complex embedding could arise from several factors, including age and lack of education. In order to separate those factors, we thus performed a fourth experiment where we tested Amazon Indians (teenagers and young adults) with little or no access to education.

### Experiment 4

#### Participants

During two field trips in 2014 and 2015, one of us (P.P.) collected behavioral data in Wariri, an isolated village of the upper Cururu region of the Munduruku main territory, located on the Anipiri River. 20 Mundurukus volunteered for this experiment: 14 teenagers (age range 10–14, mean = 12 ± 0.4) and 6 adults (age range 30–67, mean = 46 ± 6.6). As in many other villages of the Munduruku main territory, inhabitants of the Wariri village, including our volunteers, have poor and restricted access to schooling and have a very partial command of Portuguese. Munduruku language is quite impoverished in number words and Euclidean geometrical terms [27,31]. Still, previous research has shown that Mundurukus are able to grasp sophisticated concepts of number and space in an approximate and nonverbal manner [27,31,53,54].

#### Stimuli and procedure

Munduruku subjects found the adult version of the task exceedingly dull and could not be persuaded to complete it, so we substituted the shorter but analogous children’s version. The design was thus exactly the same as experiment 3 with children.

#### Results

For “irregular”, the mean error rate equaled 78 ± 3% and we observed a small but significant decrease in error rate in the second phase (ρ = -0.25, p = 0.035), indicating rote learning of the succession of positions. This ability to learn positions was confirmed by performance on the “4points” sequence, with a mean error rate of 49 ± 8%, and error rates significantly below baseline at data points 6 and 12 (p*s*< 0.013 corrected). Participants also quickly grasped the sequence “2points”, with a mean error rate of 5.6 ± 2.8%, and an error rate below the baseline from the beginning to the end of the trial (all p*s*< 0.013 corrected).

For all regular sequences, except “2crosses”, the mean error rate was significantly lower than baseline (Friedman test: all p*s*<0.003): “repeat” (across 1^st^ and 2^nd^ stage: 2.5 ± 1.2% errors; on 1^st^ stage only: 5 ± 2.8% errors), “repeat+2” (1.3 ± 0.9%; 1.7 ± 1.7% errors), “2arcs” (16.9 ± 5.9%; 23.3 ± 8.6% errors), “2squares” (26.9 ± 5.4%; 18.3 ± 6.8% errors), “4segments” (12.1 ± 2.8%; 16.1 ± 3.6% errors), “4diagonals” (59.4 ± 5.3%; 55 ± 6.2% errors) and “2rectangles” (53.1 ± 5.3%; 51.7 ± 6.3% errors). However, the mean performance in “2crosses” (78.1 ± 3.2%; 78.3 ± 5.7% errors) did not differ from baseline (F = 0.29, n.s).

We again found a positive correlation of the mean error rate with the complexity of the sequences (at all data points: Spearman’s ρ = 0.59 ± 0.02, Student t-test: t_19_ = 28, p <10^−12^; at data points 6–8: ρ = 0.51 ± 0.05, t_19_ = 11, p < 10^−5^, Fig 3, bottom panel). In this group of teenagers and adults Mundurukus, the correlation was weaker than in adults’ group (t_41_ = 3.71, p < 0.001), but slightly greater than the correlation observed in both groups of children (t_66_ = 2.00, p = 0.05).

**Fig 6:**
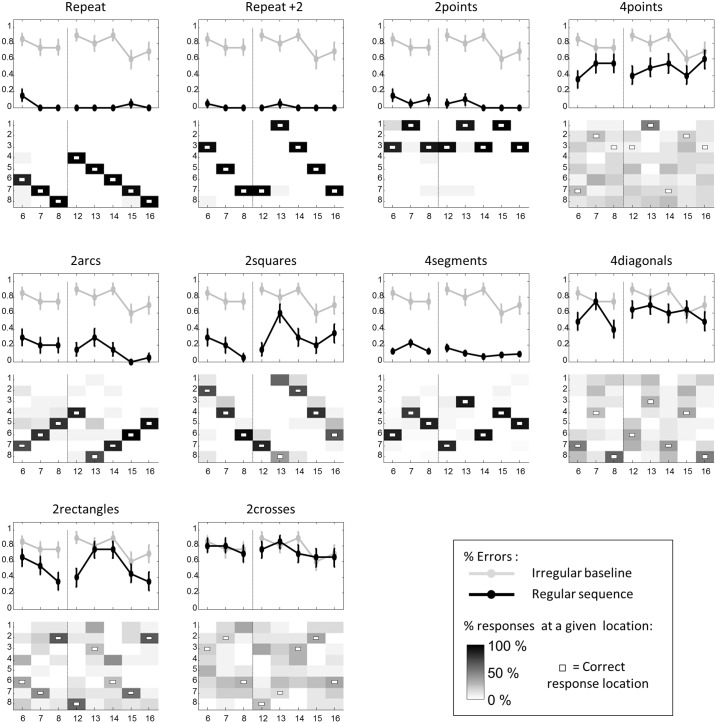
Performance of Munduruku participants in experiment 4. Same format as Fig 4.

Munduruku teenagers and adults quickly detected and used the rules +1, +2 and all axial symmetries, as shown in Fig 6 by error rates on “repeat”, “repeat+2”, and “4segments”, that were below the baseline (“repeat”: p*s*< 0.008 corrected; “repeat+2”: p*s*< 0.004 corrected; “4segments”: p*s*< 0.037 corrected except at the 15^th^ data point). The mean error rate was similar for horizontal, vertical, and oblique symmetries (vertical: 7.5 ± 3.3% errors; horizontal: 25.6 ± 8.4%; oblique: 9.4 ± 3.5% and 11.6 ± 6.6%; Friedman test for differences between the four types of symmetries: F = 3.4, n.s.). It is less clear, however, that participants were fully able to detect and use rotational symmetry, as performance with “4diagonals” was not significantly better than the baseline, but there was a tendency at data points 6, 8 and 14 (p*s*< 0.04 uncorrected).

“2arcs” and “2squares” again showed similar error patterns, suggesting that participants were able to understand both superficial and deep rules. For “2arcs”, error rate was significantly below baseline at all data points (shallower rule at points 6–8, 12, 14–16: all p*s*< 0.018 corrected; deeper rule at point 13: p = 0.031 corrected). For “2squares”, error rate was significantly below baseline at all data points except the 13^th^ and 16^th^ (all p*s*< 0.037 corrected).

On “2rectangles”, the error rate was significantly below the baseline at the 12^th^ data point (p = 0.013 corrected), indicating that some features of this three-levels sequence were grasped by participants. “2crosses” was more challenging, and the Munduruku never managed to perform better than baseline.

Interestingly, whenever there was a difference, Munduruku teenagers and adults systematically performed better than French children and worse than French adults.

#### Discussion

Munduruku teenagers and adults, although having a limited access to schooling, performed at a level close to French adults, their answers providing evidence for a quick understanding of most of the geometrical primitive rules (+1, +2 and axial symmetries), and for an ability to detect different levels of embedded regularities. Only rotational symmetry was not clearly detected, perhaps explaining their poor performance on “2crosses”. All in all, the results suggest that geometrical primitives and their combinations are available to human adults and teenagers after minimal experience, even in the absence of formal education.

### Detailed fitting of the “language of geometry” model

The above data indicate that adults quickly infer an internal representation of an unfolding geometrical sequence and use it to predict what comes next. Our experiment is predicated upon the hypothesis that this representation takes the form of a “language of thought” [34,36]: a set of precise primitive instructions that can be combined into complex expressions that faithfully capture the observed geometrical sequence. The language that we proposed supposes that “2squares” or “2arcs” can be compactly represented by two nested repetitions, and “2rectangles” or “2crosses” by 3 nested repetitions. At the same time, plausibly, it does not attribute a compact form to complex sequences where humans do no detect any specific regularity. Overall, those hypotheses seem to be correct inasmuch as complexity is a good predictor of error rates. In the present section, we go one step further and ask whether the language predicts, in a quantitative manner, why and when errors arise.

#### Model description

To predict sequence continuation behavior, we may assume that at any given moment, subjects hold on to the simplest possible hypothesis concerning the current sequence, and use this hypothesis to predict the next items. Formally, after observing the first *n* items in a sequence (hereafter the “prefix”), subjects identify the shortest expression compatible with this prefix, and then compute the continuation of this expression.

Because actual performance presented some degree of stochasticity, we also introduced what seems to be a natural source of noise in this model. Our proposal is that, as the length of an expression increases, the probability that the subject fails to properly estimate its length increases. We model this by assuming that program length is evaluated with a degree of randomness, i.e. additive Gaussian noise with standard deviation σ (constant across all sequences). Moreover, to avoid a systematically perfect performance at the last data point, we assumed that the model can only compute expressions up to a certain complexity. Here, we set a maximal capacity to K_max_ = 12. Whenever a prefix implies an expression with K > 12, the algorithm selects a response at chance.

The initial sequence (*S*) comprises the first two locations shown to the subject. From this point, the model constructs the sequence by adding one location at a time until it reaches 8, following the pseudo-algorithm below (S2 Fig):

While Number of locations < 8:

1. Consider all programs that generate sequences of 8 locations and share the prefix *S*.
2. Estimate the length of those programs, assuming that this estimation has Gaussian noise given by the free parameter σ.
3. Choose the sequence S' whose prefix matches *S* and which has complexity K(S’). If there is more than one such sequence, choose randomly between them.
4. If K(S') ≤ Kmax, then generate as a prediction the next location predicted by sequence S’; otherwise, generate a prediction at random.

#### Fits to adults’ data

**Fig 7:**
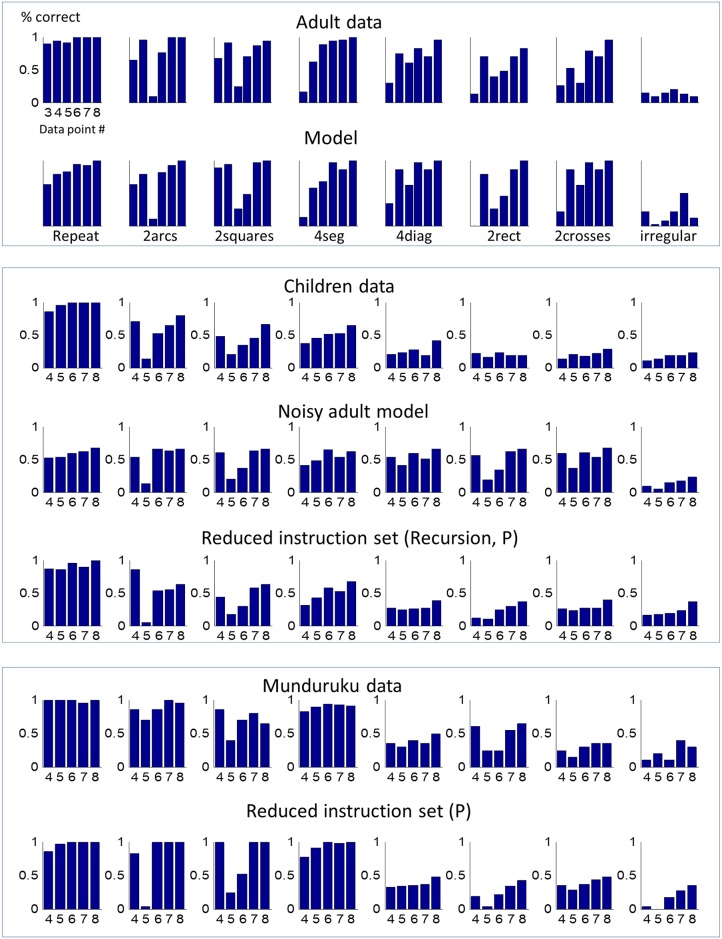
Model fits to subjects’ data. Comparisons of the correct rates exhibited in completing regular and “irregular” sequences by French adults (top), preschool children (middle) and Munduruku teenagers and adults (bottom) with the performance of our model in its full version (for French adults—top), then in a noisy version (for children—middle), and finally in a version that includes a reduced instruction set (for children—middle; and Mundurukus—bottom).

To evaluate the fit of the model to the data, we only considered the 8 sequences that were used in all groups and involved no repetition of the 8 locations. The model captured in a very robust manner, independently of parameter values, the most salient aspects of the data (Fig 7). First, it shows different degrees of performance for each sequence in agreement with the data: close to perfect performance for the repeat sequence, close to chance performance for the irregular sequences, and an intermediate progression for other sequences. The model also captures an overall trend for improving performance as the sequence progresses and, crucially, each of the local drops in performance that arise at specific points within each sequence. Indeed, the model fully accounts for the precise time points at which they occur (odd-numbered time points 3, 5 and sometimes 7, as explained in the results section).

To obtain those results, the only free parameter of the model, σ, was fit by minimizing the mean square errors (MSE) across all time points and all sequences. For each value of σ we performed 300 runs and calculated the average performance of the model for each position of the sequence. This analysis revealed a very clear minimum for σ = 2 (S3 Fig). For reference, we compared this with the MSE of the simplest possible fit, consisting in a constant level of performance, distinct for each sequence (for a total of 8 parameters). Within a broad range of noise (including the noiseless model with σ = 0) the language-of-geometry model, with its single degree of freedom, performed better than this 8-parameter model. As shown in S3 Fig, even the performance of the noiseless model, while more discrete than the real data, captures the main aspects of our results.

#### Fits to children’s data

Our model captures, without any fine parameter tuning, the nonlinear performance functions exhibited by educated adults, by assuming that they use all of the primitives available in our language. Young children or uneducated adults, however, may not master the full language of geometry.

We thus examined, first, which transformation of the model could account for the children’s data. We started by fitting the parameter σ, again using 300 independent runs for each value of σ. This analysis showed that no amount of noise could fit the data adequately. This was confirmed quantitatively (MSE for all noise values were greater than 0.3) and also from visual inspection which revealed a pattern very different from the data (Fig 7). Notably, even for the best fit, performance was massively underestimated for low-complexity sequences such as “repeat” or “2arcs”, while being massively over-estimated for high-complexity sequences such as “2rectangles” or “2crosses”.

We next examined the hypothesis that children may have additional sources of noise. Specifically, we supplied the model with an additional source of noise in the execution of each program. We assumed that the model could generate a random response (an execution error) with a probability given by a second parameter σ_2_. These two sources of noise had different effects on the simulated data. Yet, even with the inclusion of this additional noise parameter, the model still performed very poorly. Indeed, MSE values were greater than 0.18 for the full set of parameters, and the best fits were achieved with a very high execution noise, which resulted in an ability to predict the fine-grained structure of errors: the model performed in a highly unstructured manner, with a low and flat performance within and across sequences (S4 Fig).

As a third step, instead of implementing a noisy version of the full language, we assumed that children might use a subset of the language. For instance, their mental programs might lack some of the primitive instructions, or might not be able to express deep levels of nested repetitions. Based on the above results, we examined a semantically and syntactically restricted language devoid of (a) rotational symmetry primitive P (b) the ability to encode nested repetitions: while the original language allows for “repetitions of repetitions”, e.g. to encode the “2squares” sequence, we assumed that young children may only be able to encode a single level of repetition. For simplicity, we do not report here a full exploration of other possible sub-languages, which yielded no better fit.

We further assumed that the use of those two resources is probabilistic. This assumption was meant to capture variability both within subjects (e.g. a child may understand nesting and yet fail to use it on some trial) as well as between subjects (some children may not be capable of encoding nested structures). Accordingly, the original model (with single parameter σ) was supplemented with two additional parameters: *p_NEST*, the probability of using nested sequences with repetitions of repetitions, and *p_P*, the probability of using instruction P.

Our model, in this version, cannot distinguish between these alternatives. We do show below an analysis of correlations that shows that children that perform poorly in a sequence that uses the P instruction also tend to have bad performance in other sequences that use the P instruction. This suggests that, to a certain degree, there is variability in the population of young children in the degree of consolidation of their language of geometry.

To fit the data, we performed 300 independent runs of the model for a fixed level of σ = 3 and without program execution noise (σ_2_ = 0). For each run we generated two random variables that determined, with probabilities *p_NEST* and *p_P* respectively, if all sequences that used nesting or the instruction P had their complexity set to the maximum value of K = 12. This is equivalent to stating that any expression using these resources exceeds K_max_ and hence cannot be used to extract regularities (note that the alternative, which would have been to recompute all complexities K for the language with reduced instruction set, was not available because the language without the instruction P cannot generate the full set of sequences).

Varying *p_NEST* and *p_P* showed that:

1. The best performance is achieved for values *p_NEST = 0*.*14* and *p_P = 0*.*18*, which captures the children’s performance in great detail (Fig 7). These are relatively low values indicating that for the majority of children and/or trials, these resources are indeed not used to extract regularities.
2. While these values are low, a language entirely lacking these resources fits the data quite poorly, showing near-chance performance for all sequences, except for the simplest repetition of +1. (S5 Fig, Panel marked “Full Reduced Instruction set”)
3. Removing the instruction P but allowing all levels of nesting, results in a very different pattern of performance, with near-perfect performance for 4 out of the 8 sequences (S5 Fig, panel marked “No instruction P, normal nesting”)

#### Fits to Munduruku data

As with children, the noisy version of the full model could not account for the data (MSE > 0.19 for the best fit). The analysis varying *p_NEST* and *p_P* showed that:

1. The best performance is achieved for values *p_NEST = 0*.*54* and *p_P = 0*.*26* (S6 Fig). Note that both values, especially p_NEST, are higher than those obtained for young children.
2. As with the young children, a language which never uses nesting or P (i.e. with *p_NEST = 0* and *p_P = 0)* cannot account for the data, as its performance is close to chance for all sequences, except for the simplest repetition of +1. (S6 Fig, Panel Full Reduced Instructions)
3. However, compared to young children, a simplified version of the full model, removing only the instruction P but allowing all levels of nesting, results in an acceptable fit, very similar to the best fit. In fact, a plot of the value of MSE for varying probabilities (S6 Fig, color matrix) shows that the fit varies little over a broad region that includes high values of P_NEST. Thus, compared to children, simply lowering the probability of using P resulted in an accurate description of the Munduruku data (Fig 7).

## Discussion

The aim of our research was to evaluate whether the human memory for spatial sequences provides evidence for (1) an understanding of simple geometrical primitives in both educated and uneducated humans, (2) a capacity to combine those primitives into complex embedded expressions, and (3) a notion of sequence complexity based on minimum description length. We discuss those aspects in turn.

### Geometrical primitives

The findings from four experiments suggest that simple rotations (equivalent to the rules ±1, and ±2) and vertical, horizontal and oblique symmetries were all detected and quickly used by human adults with various cultural backgrounds and 5-years-old children. These results are consistent with previous work highlighting the importance of the detection of symmetries in shape perception [42,55–58] or in spatial navigation [22,59]. The primitive operations postulated in our language (±1, ±2, axial and rotational symmetries) may form part of the “core knowledge” of mathematics which is thought to be shared by all humans [28]. In Plato’s Meno (~ 380 B.C.) [60], Socrates, after interrogating an uneducated Greek slave on the area of various squares drawn in the sand, already concluded that “his soul must have always possessed [the] knowledge” (for a recent replication, see [61]). Recent evidence has confirmed the existence of core geometrical knowledge shared with other animal species and available in early infancy [21,22,24,62,63]. In particular, previous research with American and Munduruku adults and children led to the conclusion that they all exhibit a shared competence for various concepts of topology, Euclidean geometry, and basic geometrical figures [27,53].

It could be argued that the present language mixes purely geometrical properties (axial and rotational symmetries) with other arithmetic (+1, +2, +3) and abstract algebraic features (repetition). However, such a mixture is probably indispensable if we consider that geometry is a branch of mathematics concerned with questions of shape, size, relative position of figures, and the properties of space. Integers, although conceivably part of a distinct system of arithmetic, are indispensable to capture even basic geometrical concepts such as “square” or “triangle”. Numbers and space are tightly intertwined concepts, and the metaphor of numbers as a measure of space (which is the etymology of “geo-metry”) played a foundational role in the history of mathematics from Pythagoras and Euclid to Descartes and Hilbert. Mathematics is a unified discipline in which it is difficult to delineate the boundaries between geometry and other domains, and the present language reflects this simple fact.

Interestingly, previous behavioral studies also concluded that symmetries and other geometrical transformations were more difficult for Munduruku adults, Munduruku children or American children than for educated American adults [27]. The present results are in agreement with this conclusion, inasmuch as (1) axial symmetries induced more errors for Munduruku than for French adults and even more errors for French preschoolers than for Munduruku and French adults together; (2) rotational symmetry was quickly detected by French adults, but not by French preschoolers or Munduruku adults; (3) combinatorial rules that consisted in a global symmetry or rotation of a geometrical shape (e.g. in “2arcs” or “2squares”), were harder to detect for Munduruku than for French adults and even harder for French preschoolers than for Munduruku and French adults together.

One might argue that children and Mundurukus’ failure to detect rotational symmetry might be due to a greater movement distance in “4diagonals” than in “4segments”. However, this argument is made less plausible given that the successive distances between points 4, 5 and 6 of the “4segments” and “4diagonals” sequences are exactly the same, and yet the error rates are lower in “4segments” than in “4diagonals”. This observation suggests that distance had a much lesser influence, if any, than the capacity to encode rotational symmetry. It seems that rotational symmetry is inherently a more difficult mathematical concept. Nevertheless, our model simulations suggest that it was not entirely lacking in Munduruku or in children, but merely probabilistically absent in some trials and/or some children.

### Embedded expressions

Our findings also suggest that human subjects were able to detect most of the embedded expressions we used to define our visuospatial sequences. In details, all subjects easily detected simple repetition (repeat sequence) as well as the concatenation of two instructions underlying the “alternate” and “2points” sequences. Evidence for repetition with variation was also found in all groups of subjects. In particular, educated adults easily detected and encoded a systematic change in the starting point of a geometrical shape (e.g. “2squares”), or a global transformation applied to the whole shape (e.g. “2arcs”). In Munduruku, the application of these combinatorial rules was more challenging, but still led to a significant level of success. Finally, 5-years-old children performance on “4segments” tended to show that they were able to apply a repetition with a change in the starting point, and their performance on “2arcs” suggested that they were also able to apply a global symmetry.

The analysis of error patterns provided direct evidence for hierarchical embedding. Superficial rules were acquired more quickly and induced fewer errors than deeper rules. In French and Munduruku adults, the quantitative error patterns, peaking at odd-numbered time points 3, 5 and 7, were consistent with a single level of embedding for “repeat”, “repeat+2” and “alternate”; two levels of embedding for “2arcs”, “2squares”, “4segments” and “4diagonals”; and three levels of embedding for “2rectangles” and “2crosses”.

These findings thus suggest that subjects spontaneously detected the recurrence of low-level subsequences that shared a common instruction, and then combined them into hierarchically organized expressions. Those conclusions agree with those made in another domain by Kotovsky and Simon [64]: when learning a series of letters, adults first detected the periodic recurrence of some letters, then used it to infer higher-order rules. These authors showed that the postulation of a hierarchical organization of rules was crucial in capturing the subjects’ behavior.

Moreover, the good performance achieved by subjects on time points 6, 7 and 8, even before the entire sequence had been presented, indicates that they quickly inferred an internal representation of the sequence and used it to predict the next locations. This is consistent with works led by Restle in the 70’s [6,65–67], in which he showed that adults, when asked to anticipate or track the positions of a series of flashes, easily grouped consecutive items in what he called “runs” (e.g. 1-2-3, where numbers refer to ordinal positions) or “trills” (e.g. 1-2-1-2) and used these regularities to predict the next locations. Restle’s research showed that adults progressively learned how to combine “runs” and “trills” by building a mental tree structure that encoded the sequence of flashes they had been presented with [6,65–67].

Our experiments 2 and 3 showed that 5-years-old children experienced difficulties in understanding complex sequences, either involving rotational symmetry or the use of multiple nested calls to the “repeat” instruction. The latter finding, using temporal spatial sequences, can be related to research on the perception of static spatial patterns in childhood [18]. Using fractals, Martins et al. tested 7–8 years-old and 9–10 years-old’s ability to represent recursive rules (generating additional hierarchical levels) versus iterative rules (inserting additional items within an existing hierarchical level). They concluded that all children could detect iterative rules, but only fourth graders (9–10 years-old) were able to detect recursive rules.

Collectively, those results suggest an influence of age or education level on the ability to understand hierarchically organized geometrical rules. Crucially, however, Munduruku teenagers and adults, who lacked school-based education, performed better than children on sequences with 2 or 3 levels of embedding. Indeed, their results could be accounted for solely by the absence of rotational symmetry. This finding suggests that schooling may not be necessary for the development of the ability to understand nested rules. With age, it seems that a geometrical language with embedding arises even in the absence of formal schooling. In fact, even in young children, the failure with complex sequences need not be due to a lack of understanding of nested structures, but could arise from limitations in working memory, inasmuch as the detection of such sequences requires a visual memory span of at least four. Indeed, even in the absence of any regularity, children failed in memorizing an irregular sequence of length 4, suggesting that their visuo-spatial memory span was below this critical value. Further work will be needed to assess whether children would succeed with nested structures if the working memory load was alleviated.

### Minimal description length as a predictor of spatial memory

We defined the theoretical complexity of a sequence as the length of the shortest expression capable of generating it (following Kolmogorov’s ideas [44] and the minimum description length principle [46]). In educated adults, this measure of complexity was an excellent predictor of the mean error rate (Fig 7), suggesting that it provides a good approximation of the internal representational complexity of spatial sequences. Such a relationship is in accordance with previous works on conceptual learning. Feldman [51], following earlier work by Shepard, Hovland and Jenkins [68], showed that the description length of Boolean concepts captured the difficulty that humans experienced in learning these concepts. Minimal description length was also successfully used by Bradmetz and Mathy [47] to model the response times of human adults in a task requiring conceptual learning of classification rules. Moreover, Mathy and Feldman [48] found that minimal description length was positively correlated with the memorability of a sequence of digits. Our findings confirm that minimal description length provides a reasonable approach to adult sequence learning capacity. For children and Munduruku subjects, a language with reduced instruction set led to similar conclusions.

In passing, we note that there is a near-complete equivalence between the present Kolmogorov-complexity approach and Bayesian model-selection approaches to sequence learning [40,51]. In [51], internal models are first assigned a prior probability proportional to their complexity, and then this probability is increased or decreased depending on how well each model accounts for the incoming data or, on the contrary, generates a prediction error. This is tantamount to selecting the simplest program that accounts for the observed data, as we do here. In [40], the multi-sensory representations of visual or auditory sequences of locations around a circle were modeled as computer programs. These programs were formalized using a probabilistic context-free grammar, and learnt via Bayesian inference. Similar to our work, the prior distribution favored the simplest, shortest programs. We also note that the spatial language used in [40] was closely related to ours (including instructions “next” and “prev” similar to our +1 and -1, loops and recursion). Crucially, however, it lacked geometrical primitives such as horizontal or vertical symmetry that the present work suggests are essential to capture the organization of more complex spatial sequences.

We end by pointing to several limitations of this work. Our model rests on a narrow language that should not be taken as a complete description of “core geometry”. Many additional primitives, both geometrical (e.g. right-angle, parallelism, triangle, distance…) and non-geometrical (e.g. integer sequences) would need to be added to capture the full range of core human intuitions [27]. A particularity of our language resides in the fact that each location is defined relatively to preceding ones thanks to the application of a given geometrical rule. While this choice allowed for a simple definition of complexity, it also resulted in the fact that some simple geometrical shapes could not be easily captured. For instance, in the current language, a circle or an equilateral triangle could not be described. In the future, the present methodology should be extended in order to fully characterize the range of sequences, shapes and scenes that humans readily consider as “geometrically simple”.

## Supporting information

S1 Fig Comparison of different potential predictors of error rates. For each sequence, the y axis represents the mean error rate of French adults, and the x axis the sequence complexity, as measured by complexity computed in the full language (top), complexity computed in a degraded language including only the rules ±1, ±2, ±3, +4 and repetitions without symmetries (middle), and the normalized jump length of a sequence (bottom). Regression lines are also plotted and Spearman’s correlation coefficients are displayed. The middle and bottom plots reveal clear outliers.
(TIF)

S2 Fig Model description. Starting from prefix prf, the algorithm lists all possible sequences and their associated programs P in our language, computes their associated complexity K(P) introducing Gaussian noise, then chooses the program that minimizes K(P), and completes the prefix prf with the next location either defined by P if K(P) does not exceed the complexity threshold K_max_, or chosen randomly if K(P) is greater than K_max_.
(TIF)

S3 Fig Fit of the data for varying values of *σ*. Even for low values of noise, the model identifies the pattern of performance throughout the sequences (compare to the top panel showing the data for adults).
(TIF)

S4 Fig Fit of children’s data using a noisy version of the adult geometrical language. The top panel shows the observed performance in preschoolers for each sequence. The matrix in the middle shows the minimum mean square error (MMSE), i.e. the quality of the fit, as a function of the amplitude of the noise in encoding *σ* and execution *σ2*. Even the best-fitting model with these two noise parameters (bottom) shows a performance very different to the data, with almost equal performance for all sequences.
(TIF)

S5 Fig Comparison of different fits of children’s data. Children data (top panel) is not well described by the adult geometrical model (second panel from the top). The matrix in the center shows the quality of the fit as a function of the probability p_P of having the P instruction (+4) and the probability p_Nest of having Nest > 1 in the language. The data is best captured by a model with low values of P and Nest >1 (third panel). However, when making these probabilities equal to zero (fourth panel) the model describes the data very poorly. Similarity, a model allowing for full nesting while fitting p_P (fifth panel) inappropriately predicts near-perfect performance for the first four sequences.
(TIF)

S6 Fig Comparison of different fits of Mundurucus’ data. Mundurucus’ data (top panel) is not well described by the full model (second panel from the top). The image in the center shows the quality of the fit as a function of the probability of having the P instruction (+4) and the probability of having nested repetitions in the language. The data is best captured by a model with low but non-zero values of p_P and p_Nest (third panel). Letting these probabilities equal to zero (fourth panel) leads to a model that describes the data very poorly. A model with full nesting, fitting only p_P (fifth panel), results in a fit comparable to the best fit.
(TIF)

S1 Text Language of geometry. Description of the programming language used in this study.
(PDF)
