# Supplementary figures and images for: The language of geometry: Fast comprehension of geometrical primitives and rules in human adults and preschoolers
Source: PLoS Comput Biol. 2017 Jan 26;13(1):e1005273. doi: 10.1371/journal.pcbi.1005273 (PMC5305265; doi:10.1371/journal.pcbi.1005273)

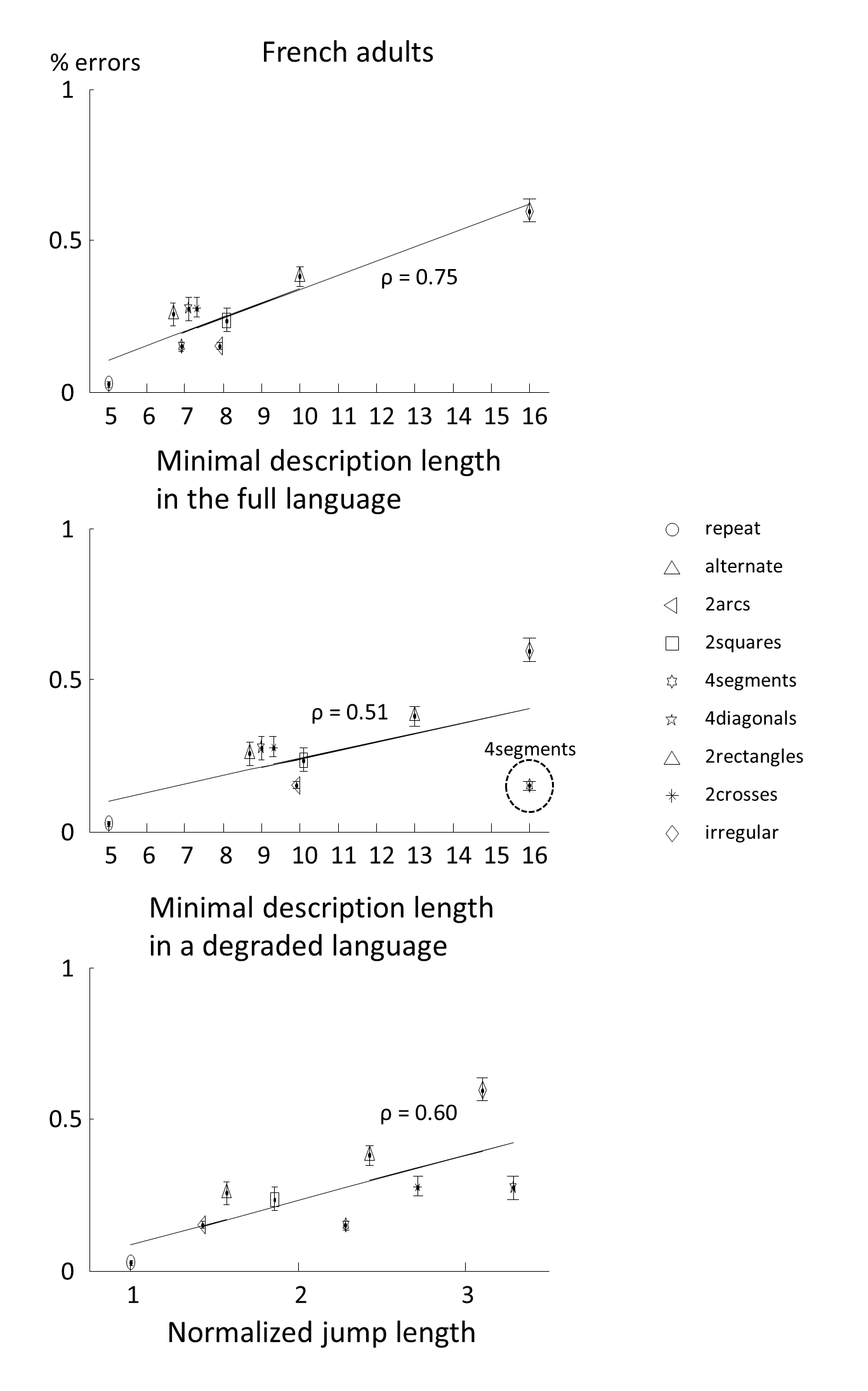

Supplement: S1 Fig — For each sequence, the y axis represents the mean error rate of French adults, and the x axis the sequence complexity, as measured by complexity computed in the full language (top), complexity computed in a degraded language including only the rules ±1, ±2, ±3, +4 and repetitions without symmetries (middle), and the normalized jump length of a sequence (bottom). Regression lines are also plotted and Spearman’s correlation coefficients are displayed. The middle and bottom plots reveal clear outliers. (TIF) [file pcbi.1005273.s001.tif]

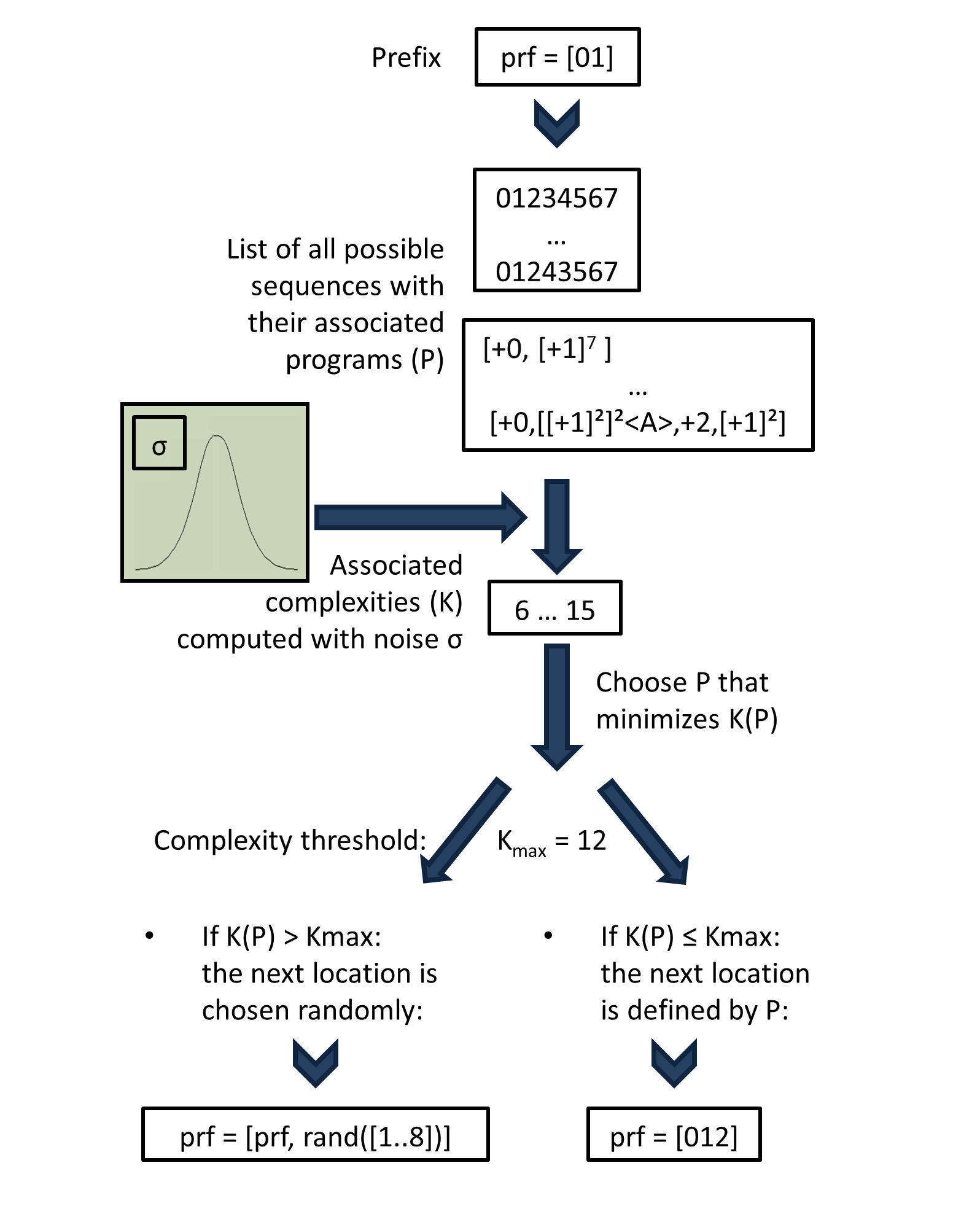

Supplement: S2 Fig — Starting from prefix prf, the algorithm lists all possible sequences and their associated programs P in our language, computes their associated complexity K(P) introducing Gaussian noise, then chooses the program that minimizes K(P), and completes the prefix prf with the next location either defined by P if K(P) does not exceed the complexity threshold Kmax, or chosen randomly if K(P) is greater than Kmax. (TIF) [file pcbi.1005273.s002.tif]

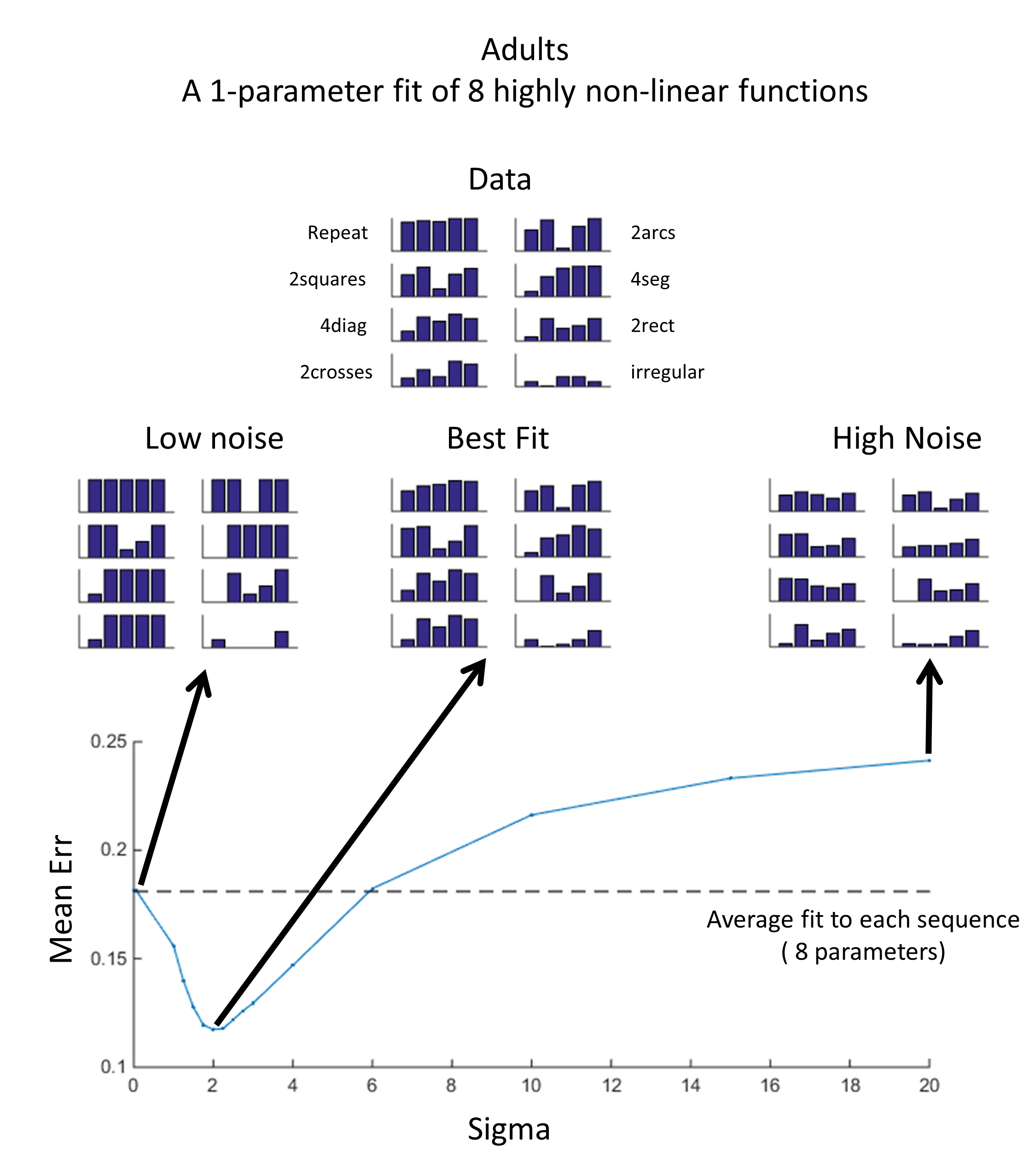

Supplement: S3 Fig — Even for low values of noise, the model identifies the pattern of performance throughout the sequences (compare to the top panel showing the data for adults). (TIF) [file pcbi.1005273.s003.tif]

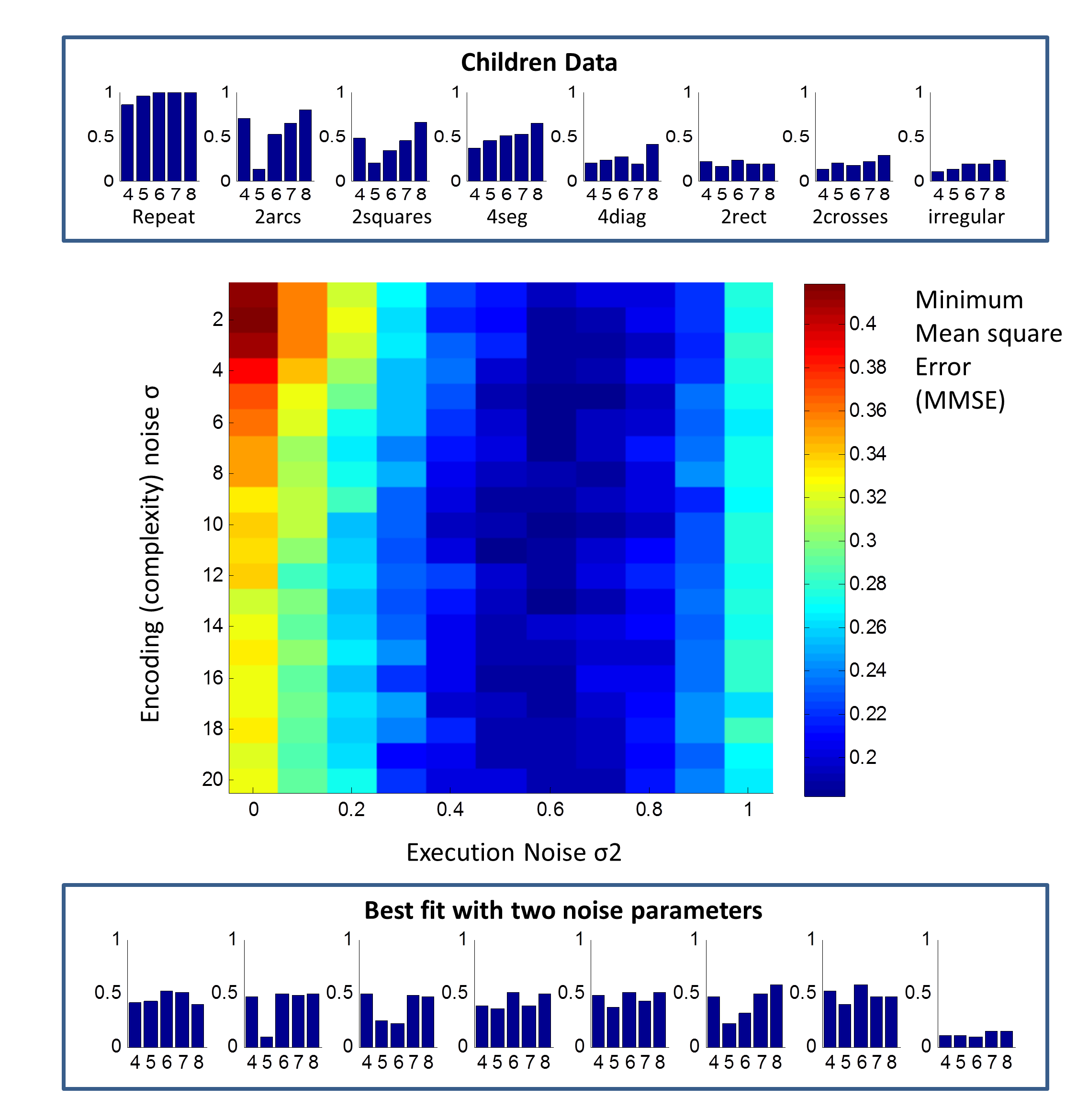

Supplement: S4 Fig — The top panel shows the observed performance in preschoolers for each sequence. The matrix in the middle shows the minimum mean square error (MMSE), i.e. the quality of the fit, as a function of the amplitude of the noise in encoding σ and execution σ2. Even the best-fitting model with these two noise parameters (bottom) shows a performance very different to the data, with almost equal performance for all sequences. (TIF) [file pcbi.1005273.s004.tif]

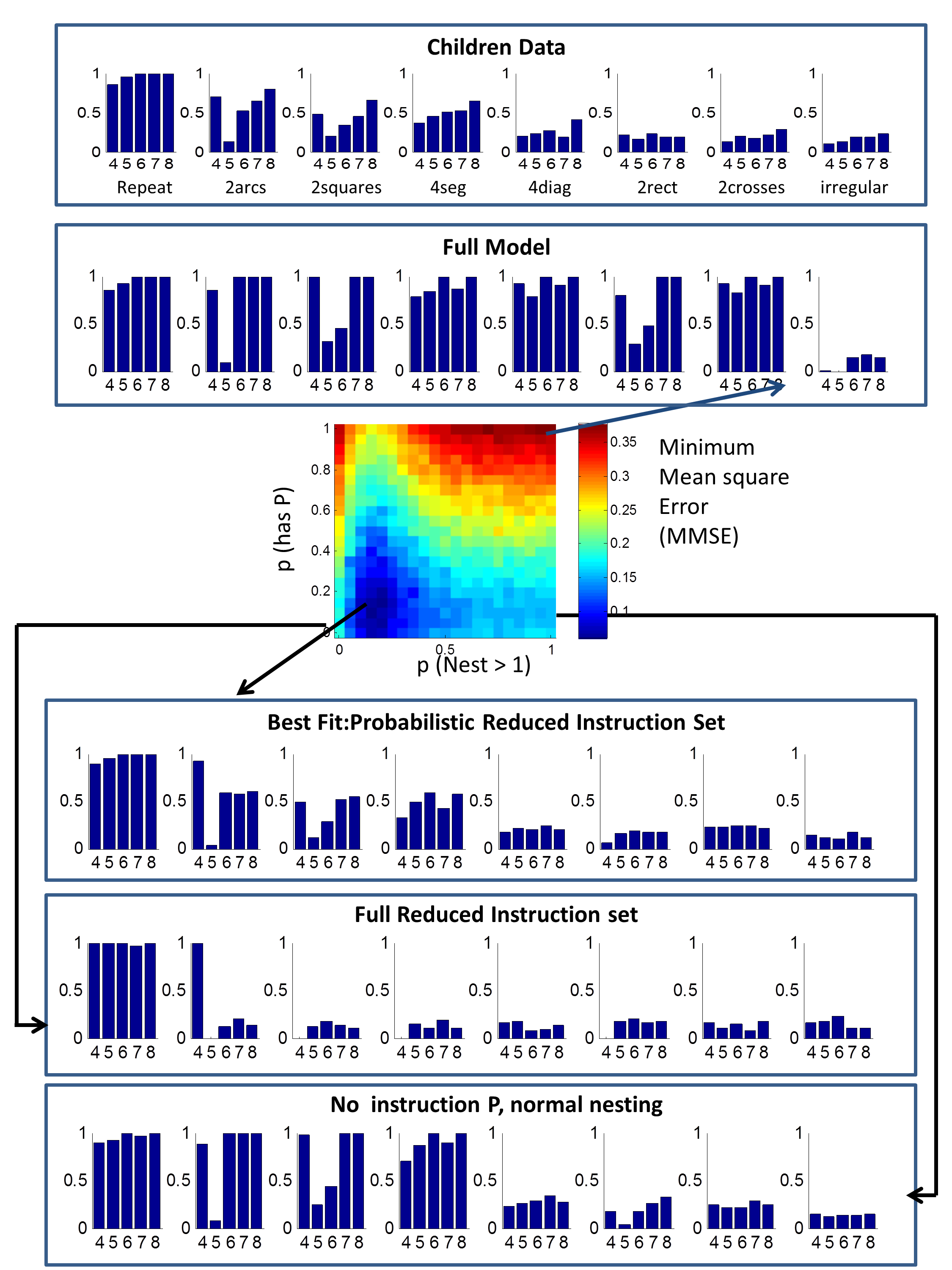

Supplement: S5 Fig — Children data (top panel) is not well described by the adult geometrical model (second panel from the top). The matrix in the center shows the quality of the fit as a function of the probability p_P of having the P instruction (+4) and the probability p_Nest of having Nest > 1 in the language. The data is best captured by a model with low values of P and Nest >1 (third panel). However, when making these probabilities equal to zero (fourth panel) the model describes the data very poorly. Similarity, a model allowing for full nesting while fitting p_P (fifth panel) inappropriately predicts near-perfect performance for the first four sequences. (TIF) [file pcbi.1005273.s005.tif]

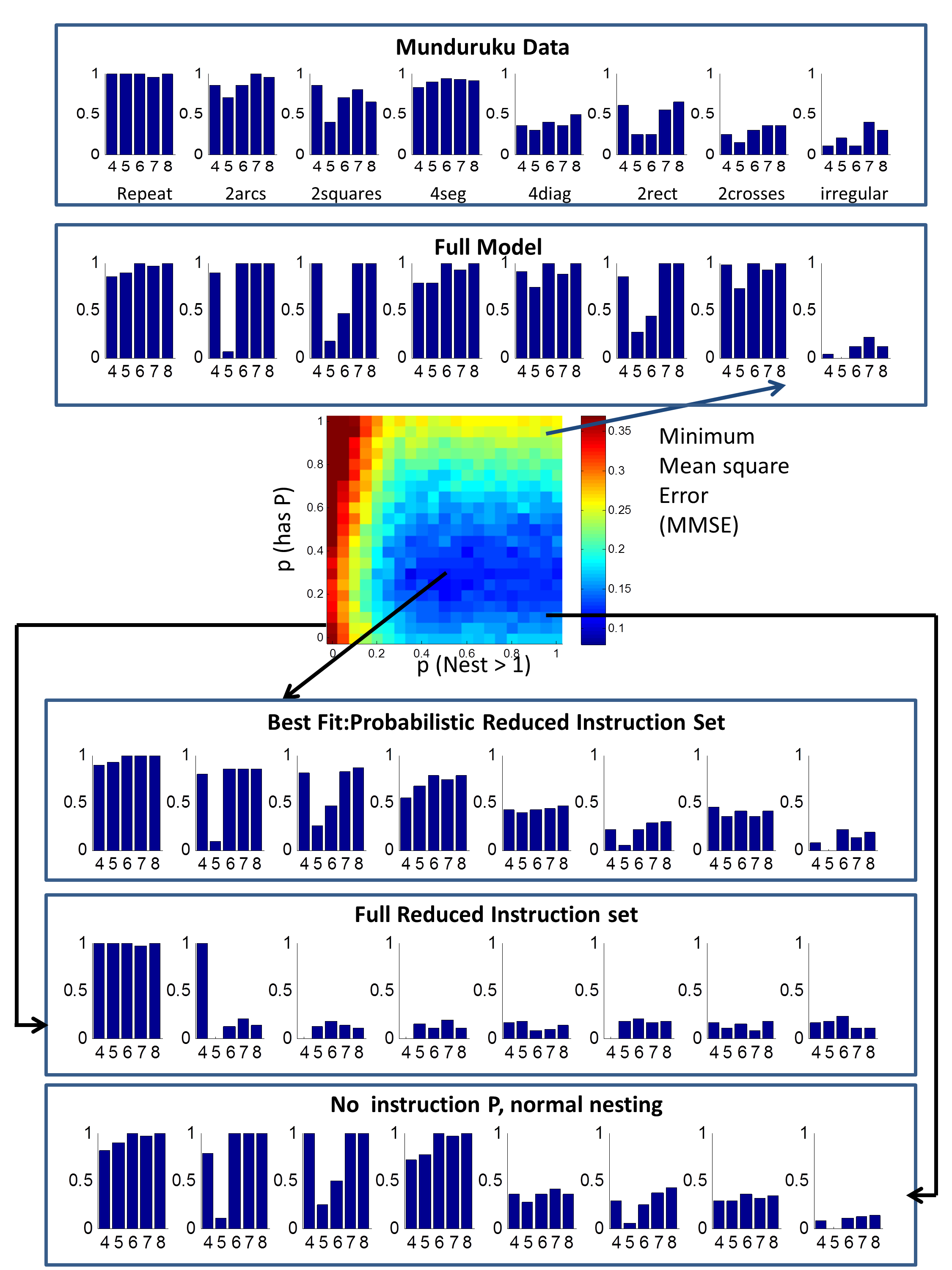

Supplement: S6 Fig — Mundurucus’ data (top panel) is not well described by the full model (second panel from the top). The image in the center shows the quality of the fit as a function of the probability of having the P instruction (+4) and the probability of having nested repetitions in the language. The data is best captured by a model with low but non-zero values of p_P and p_Nest (third panel). Letting these probabilities equal to zero (fourth panel) leads to a model that describes the data very poorly. A model with full nesting, fitting only p_P (fifth panel), results in a fit comparable to the best fit. (TIF) [file pcbi.1005273.s006.tif]
