# Supplementary material for: The language of geometry: Fast comprehension of geometrical primitives and rules in human adults and preschoolers
Source: PLoS Comput Biol. 2017 Jan 26;13(1):e1005273. doi: 10.1371/journal.pcbi.1005273 (PMC5305265; doi:10.1371/journal.pcbi.1005273)
Supplement: S1 Text — Description of the programming language used in this study. (PDF) [file pcbi.1005273.s007.pdf]

Language of Geometry – Syntax, Semantics and Complexity

May 4, 2016

We define a simple programming language called *language of geometry*, $\mathcal{L}G$, whose programs describe mappings $\Sigma \to \Sigma^+$, for $\Sigma = \{0, \ldots, 7\}$. An expression in this programming language (hereafter called a program p), on input $n \in \Sigma$ (regarded as a natural number), is represented as $p(n)$, and it is a nonempty string formed by symbols in Σ. The strings in Σ^+ should be better thought to represent paths along the points in a circle, as depicted in Figure 1. $\mathcal{L}G$ will contain instructions that can navigate the circle from one point to another and then to another and so on, jumping from one point to another a fixed number of steps, both clockwise or counterclockwise, and using the reflection over some axes. It also contains some structures to repeat certain paths.

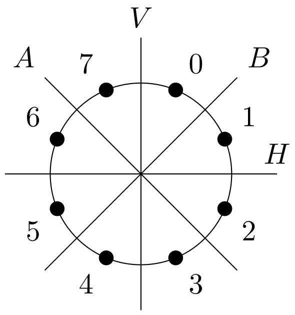

Figure 1: The symbols of Σ around a circle, and the reflection axes.

We first define the syntax of $\mathcal{L}G$ and then introduce its formal semantics.

Syntax. The set of *instructions* is defined as

$$\mathcal{I} = \{+0, +1, +2, +3, -1, -2, -3, P, A, B, H, V\}.$$

Any *program* p is a list of the form $[p_1, p_2, \ldots, p_\ell]$, where each p_i is either an instruction in $\mathcal{I}$, or an expression of the form q^s, or $q^s\langle i\rangle$, or $q^s\{i\}$, where where q is a program, s is the decimal representation of some positive integer, and $i \in \mathcal{I}$.

Semantics. The idea is that any program p of $\mathcal{L}G$ on input n describes a string in Σ^+. The instructions in $\mathcal{I}$ will allow us to navigate from a given point n into another, and the expressions of the form q^s, or $q^s\langle i\rangle$, or $q^s\{i\}$ will represent three kind of loops, which will allow us to repeat a given pattern along the circle.

Let $p = [p_1, \ldots, p_\ell]$ be a program of $\mathcal{L}G$ and let n be a number in $\{0, \ldots, 7\}$. We define the string $p(n) \in \Sigma^+$ recursively in the complexity of p:

- If $\ell = 1$ and $p_1 \in \mathcal{I}$ then $p(n)$ is defined as follows:
  - $[+0](n) \stackrel{\text{def}}{=} n$ (no change)
  - $[+1](n) \stackrel{\text{def}}{=} n + 1 \mod 8$ (move 1 clockwise)
  - $[-1](n) \stackrel{\text{def}}{=} n - 1 \mod 8$ (move 1 counterclockwise)
  - $[+2](n) \stackrel{\text{def}}{=} n + 2 \mod 8$ (move 2 clockwise)
  - $[-2](n) \stackrel{\text{def}}{=} n - 2 \mod 8$ (move 2 counterclockwise)
  - $[+3](n) \stackrel{\text{def}}{=} n + 3 \mod 8$ (move 3 clockwise)
  - $[-3](n) \stackrel{\text{def}}{=} n - 3 \mod 8$ (move 3 counterclockwise)
  - $[P](n) \stackrel{\text{def}}{=} 4 + n \mod 8$ (move to the opposite point)
  - $[H](n) \stackrel{\text{def}}{=} 3 - n \mod 8$ (reflect over H)
  - $[V](n) \stackrel{\text{def}}{=} 7 - n \mod 8$ (reflect over V)
  - $[A](n) \stackrel{\text{def}}{=} 5 - n \mod 8$ (reflect over A)
  - $[B](n) \stackrel{\text{def}}{=} 1 - n \mod 8$ (reflect over B)
- If $\ell = 1$ and $p_1 \notin \mathcal{I}$ then $p(n)$ is recursively defined as follows:
  - If $p_1 = q^s$, then
    $$p(n) \stackrel{\text{def}}{=} q(n_0) \dots q(n_{s-1}),$$
    where $n_0 \stackrel{\text{def}}{=} n$ and n_{k+1} is the last symbol of $q(n_k)$. In other words, q^s on input n represents a path given by:

    start in n and repeat s times the path represented by q.
  - if $p_1 = q^s\langle i \rangle$ and $q(n) = x_1^0 \dots x_k^0$ (for $x_j^k \in \Sigma$), then
    $$p(n) \stackrel{\text{def}}{=} x_1^0 \dots x_k^0\ x_1^1 \dots x_k^1 \dots x_1^{s-1} \dots x_k^{s-1},$$
    where $x_j^{k+1} \stackrel{\text{def}}{=} [i](x_j^k)$. In other words, $q^s\langle i \rangle$ on input n represents a path given by:

    follow the path represented by q starting in n. Say this is path σ_0. Then construct a path σ_1 which consists of applying i to each single point of σ_0. Repeat this until you reach σ_{s-1}
  - if $p_1 = q^s\{i\}$ and $q(n) = x_1 \dots x_k$ (for $x_j \in \Sigma$), then
    $$p(n) \stackrel{\text{def}}{=} q(n_0)\ q(n_1) \dots q(n_{s-1}),$$
    where $n_0 \stackrel{\text{def}}{=} n$ and $n_{k+1} \stackrel{\text{def}}{=} [i](n_k)$. In other words, $q^s\{i\}$ on input n represents a path given by:

    repeat s times the following: starting in n, follow the path represented by q, and set $n := [i](n)$.
- Suppose $\ell > 1$ and let m be the last symbol of $p_1(n)$. We define
  $$p(n) \stackrel{\text{def}}{=} p_1(n)\ [p_2, \dots, p_\ell](m).$$

Examples. Let us see some examples:

- $[+1, -1](1) = 21$
- $[+0]^4(3)$ is the concatenation of $[+0](3)$ (which is 3), $[+0](3)$, $[+0](3)$ and $[+0](3)$, i.e. $[+0]^4(3) = 3333$.
- $[+1]^4(0)$ is the concatenation of $[+1](0)$ (which is 1), $[+1](1)$ (which is 2), $[+1](2)$ (which is 3) and $[+1](3)$ (which is 4), i.e. $[+1]^4(0) = 1234$.
- $[+1, -1]^3(1)$ is the concatenation of $[+1, -1](1)$ (which is 21), $[+1, -1](1)$ and $[+1, -1](1)$, i.e. $[+1, -1]^3(1) = 21212121$.
- $[[+1]^4, [-2, [+1]^3]^2](0)$ is the concatenation of $[+1]^4(0)$ (which is 1234) and $[-2, [+1]^3]^2(4)$. Now this one last is the concatenation of $[-2, [+1]^3](4)$ (which is 2345) and $[-2, [+1]^3](5)$ (which is 3456). In total we have $[[+1]^4, [-2, [+1]^3]^2](0) = 123423453456$.
- $[[[+1]^4]^3\langle +1\rangle](0)$ is the concatenation of $[[+1]^4](0)$ (which is 1234), $[+1](1)$ $[+1](2)$ $[+1](3)$ $[+1](4)$ (which is 2345), and $[+1](2)$ $[+1](3)$ $[+1](4)$ $[+1](5)$ (which is 3456). In total, we have $[[[+1]^4]^3\langle +1\rangle](0) = 123423453456$.
- $[[[+1]^4]^2\langle B\rangle](0)$ is the concatenation of $[[+1]^4](0)$ (which is 1234) and $[B](1)$ $[B](2)$ $[B](3)$ $[B](4)$ (which is 0765). In total we have $[[[+1]^4]^2\langle B\rangle](0) = 12340765$.
- $[[[V]^2]^4\{-1\}](3)$ is the concatenation of $[[V]^2](3)$ (which is 43), $[[V]^2](n_1)$ (for $n_1 = [-1](3) = 2$, which is 52), $[[V]^2](n_2)$ (for $n_2 = [-1](n_1) = 1$, which is 61), and $[[V]^2](n_3)$ (for $n_3 = [-1](n_2) = 0$, which is 70). In total we have $[[[V]^2]^4\{-1\}](3) = 43526170$.

Complexity. We define a Kolmogorov complexity relative to $\mathcal{L}G$, that is, a measure of succinctness of a given string $\sigma \in \Sigma^+$ when the language of description is $\mathcal{L}G$. Those strings which admit a small program describing it will have small complexity, and those strings which can only be described by large programs will have high complexity.

Before defining formally the notion of complexity, we need to formalize the idea of *size* of a program. Let $p = [p_1, \ldots, p_\ell]$ be a program. With $|p|$ we denote the *size* of p . Roughly, $|p|$ is the number of letters needed to write down p. Formally, $|p|$ is defined as follows:

- If $\ell = 1$ and $p_1 \in \mathcal{I}$ then $|p| \stackrel{\text{def}}{=} 2$.
- If $\ell = 1$ and $p_1 \notin \mathcal{I}$ then $|p|$ is recursively defined as follows:
  - If $p_1 = q^s$, then $|p| \stackrel{\text{def}}{=} |q| + \lceil \log s \rceil$
  - if $p_1 = q^s\langle i\rangle$ or $p_1 = q^s\{i\}$, then $|p| \stackrel{\text{def}}{=} |q| + \lceil \log s \rceil + |[i]|$
- If $\ell > 1$ then $|p| \stackrel{\text{def}}{=} |p_1| + |[p_2, \ldots, p_\ell]|$

Let $\sigma \in \Sigma^+$. We define the *complexity* of σ, notated $K(\sigma)$, as the size of the smaller program which on input 0 describes σ, that is,

$$K(\sigma) \stackrel{\text{def}}{=} \min\{|p| \colon p(0) = \sigma\}.$$

In Figure 2 we show the complexity of some strings σ and all programs p such that $p(0) = \sigma$ and $|p| = K(\sigma)$.

**Repeat+1**
01234567
$K = 5$
$[+0, [+1]^7]$
$[[+0]^8\langle+1\rangle]$
$[[+0]^8\{+1\}]$

**Repeat+2**
02460246
$K = 5$
$[+0, [+2]^7]$
$[[+0]^8\langle+2\rangle]$
$[[+0]^8\{+2\}]$

**Alternate**
0213243546576071
$K = 7$
$[[+0, +2]^8\langle+1\rangle]$
$[[+0, +2]^8\{+1\}]$

**2points**
02020202
$K = 7$
$[[+0, +2]^4\langle+0\rangle]$
$[[+0, +2]^4\{+0\}]$

**2arcs**
01237654
$K = 8$
$[[+0, [+1]^3]^2\langle V\rangle]$
$[[[+0]^4\langle+1\rangle]^2\langle V\rangle]$
$[[[+0]^4\{+1\}]^2\langle V\rangle]$

**2squares**
02467135
$K = 8$
$[[+0, [+2]^3]^2\langle-1\rangle]$
$[[[+0]^4\langle+2\rangle]^2\langle-1\rangle]$
$[[[+0]^4\{+2\}]^2\langle-1\rangle]$
$[[+0, [+2]^3]^2\{-1\}]$
$[[[+0]^4\langle+2\rangle]^2\{-1\}]$
$[[[+0]^4\{+2\}]^2\{-1\}]$
$[[+0, [+2]^3]^2\{V\}]$
$[[[+0]^4\langle+2\rangle]^2\{V\}]$
$[[[+0]^4\{+2\}]^2\{V\}]$

**4points**
02360236
$K = 11$
$[[+0, +2, +1, +3]^2\langle+0\rangle]$
$[[+0, +2, +1, B]^2\langle+0\rangle]$
$[[+0, +2, A, +3]^2\langle+0\rangle]$
$[[+0, +2, A, B]^2\langle+0\rangle]$
$[[+0, +2, +1, +3]^2\{+0\}]$
$[[+0, +2, +1, B]^2\{+0\}]$
$[[+0, +2, A, +3]^2\{+0\}]$
$[[+0, +2, A, B]^2\{+0\}]$

**4segments**
01726354
$K = 7$
$[[+0, B]^4\{-1\}]$

**4diagonals**
04152637
$K = 7$
$[[+0, P]^4\langle+1\rangle]$
$[[+0, P]^4\{+1\}]$

**2rectangles**
05416327
$K = 10$
$[[[+0, -3]^2\langle P\rangle]^2\langle-2\rangle]$
$[[[+0, A]^2\langle P\rangle]^2\langle-2\rangle]$
$[[[+0, -3]^2\{P\}]^2\langle-2\rangle]$
$[[[+0, A]^2\{P\}]^2\langle-2\rangle]$
$[[[+0, -3]^2\langle P\rangle]^2\{-2\}]$
$[[[+0, -3]^2\{P\}]^2\{-2\}]$

**2crosses**
04512673
$K = 7$
$[[+0, P]^4\langle-3\rangle]$
$[[+0, P]^4\{-3\}]$

Figure 2: Some strings, its complexity and all minimal programs describing it.
